# A new family of extraterrestrial amino acids in the Murchison meteorite

**DOI:** 10.1038/s41598-017-00693-9

**Published:** 2017-04-04

**Authors:** Toshiki Koga, Hiroshi Naraoka

**Affiliations:** 1 Department of Earth and Planetray Sciences, Kyushu University, 744 Motooka, Nishi-ku, Fukuoka 819-0395 Japan; 2 Research Center for Planetary Trace Organic Compounds, Kyushu University, 744 Motooka, Nishi-ku, Fukuoka 819-0395 Japan

## Abstract

The occurrence of extraterrestrial organic compounds is a key for understanding prebiotic organic synthesis in the universe. In particular, amino acids have been studied in carbonaceous meteorites for almost 50 years. Here we report ten new amino acids identified in the Murchison meteorite, including a new family of nine hydroxy amino acids. The discovery of mostly C_3_ and C_4_ structural isomers of hydroxy amino acids provides insight into the mechanisms of extraterrestrial synthesis of organic compounds. A complementary experiment suggests that these compounds could be produced from aldehydes and ammonia on the meteorite parent body. This study indicates that the meteoritic amino acids could be synthesized by mechanisms in addition to the Strecker reaction, which has been proposed to be the main synthetic pathway to produce amino acids.

## Introduction

The extraterrestrial synthesis of amino acids is an intriguing discussion concerning the chemical evolution for the origins of life in the universe, because amino acids are fundamental building blocks of terrestrial life. The extraterrestrial amino acid distribution has been extensively examined using carbonaceous chondrites, which are the most chemically primitive meteorites containing volatile components such as water and organic matter, particularly the Murchison meteorite since it’s fall in 1969. The Murchison meteorite is classified as a CM2 (Mighei-type) chondrite, moderately altered by aqueous activity on the parent body (e.g. ref. 1). Currently, a total of 86 amino acids have been identified in the Murchison meteorite as α, β, γ and δ amino structures with a carbon number between C_2_ and C_9_ including dicarboxyl and diamino functional groups^2–4^. Although the presence of C_10_ amino acids has been suggested^5^, definitive C_10_ amino acid identification was not assigned to the molecular structure due to lack of the appropriate standards. The concentration and structural diversity of amino acids generally increase after hydrolysis of the water extract of the CM chondrites^6^, even though some CR chondrites yielded more free amino acids than hydrolyzed amino acids^5^. The hydrolyzed amino acids are present as their precursors and the molecular occurrence of precursors relates to the sources and formation pathways of extraterrestrial amino acids^7, 8^. It is generally considered that meteoritic amino acids could be formed in the meteorite parent bodies by the Strecker reaction, in which aldehyde or ketone reacts with cyanide and ammonia followed by hydrolysis to produce α-amino acid^9^. However, the Strecker reaction produces only α-amino acids (i.e. bearing amino and carboxyl group at the same carbon), and cannot explain the formation of other amino acids (β, γ and δ structures). It has been proposed that β-alanine could be formed by Michael addition of NH_3_ to cyanoacetylene^10^ and that γ- and δ-amino acids could be formed by hydrolysis of 5-membered and 6-membered lactams, respectively^9^. However, comprehensive formation mechanisms of extraterrestrial amino acids are not well understood.

Even though the Murchison meteorite has been studied for the occurrence of amino acids for almost 50 years, this study has revealed the presence of ten new amino acids, including a new family of nine hydroxy C_3_ and C_4_ amino acids. These new findings will expand our knowledge concerning the formation mechanism of meteoritic amino acids.

## Results

### Newly discovered amino acids in Murchison

Thirty amino acids between C_2_ and C_6_ were identified in the hydrolyzed samples of the water extract and the extract residue of the Murchison meteorite (see the Supplementary Information, SI, for the detailed analytical methods and quantification) without consideration of their enantiomers (Table 1). Many other amino acids (especially for C_5_-C_7_ amino acids) were reported from the Murchison meteorite^5, 11^, but could not be detected in this study due to the lack of appropriate standards. Glycine was the most abundant amino acid (~3 ppm in total), consistent with previous studies^6, 11^, even though α-aminoisobutyric acid (AIBA) or isovaline is sometimes the most abundant in the Murchison meteorite e.g. ref. 5. Such a distribution difference shows the heterogeneity of amino acids in the meteorite. The second most abundant amino acid was alanine (1.7 ppm) with a relative enrichment of L-alanine (Table 1). Because L-alanine is a common proteinogenic amino acid of life, and because small amounts of L-alanine, L-serine, L-threonine and L-leucine in addition to glycine were detected in the procedural blank (see SI), the L-predominance observed in this study may be due to contamination in the terrestrial environment since the fall of the Murchison meteorite in 1969. However, the L-preference of proteinogenic amino acids such as alanine and aspartic acid in the Murchison and Tagish Lake meteorites has been proposed to be indigenous^12, 13^. Amino acids such as AIBA, β-alanine and isovaline were also present in relatively high concentrations (0.97, 1.7 and 1.7 ppm, respectively) in this study. These non-proteinogenic amino acids have been reported as indigenous amino acids in the Murchison meteorite as well as other CM chondrites by many previous studies^11, 14–16^.

**Table 1:** Amino acids identified in the Murchison meteorite and the experimental products.

Carbon number	Position of amino group	Amino acid	Murchison (ppb) (n = 2)			Experiments (ppm)		m/z**
			H_2_O extract hydrolyzed	Residue hydrolyzed	Total (Gly = 100)	HCHO/CH_3_CHO/NH_3_ (Gly = 100) (n = 2)	HCHO/CH_3_CHO/CH_2_(OH)CHO/NH_3_ (Gly = 100) (n = 3)	
2	α	Glycine	1373 ± 143	2192 ± 82	3566 ± 165 (100)	237 ± 26 (100)	288 ± 42 (100)	126
3	α	D-Alanine	318 ± 59	252 ± 18	570 ± 62 (16)	14 ± 2 (5.9)	159 ± 26 (55)	140
	α	L-Alanine	398 ± 74	732 ± 8	1130 ± 75 (32)	14 ± 2 (5.9)	163 ± 27 (57)	140
	α	Sarcosine	201 ± 26	97 ± 5	298 ± 26 (8.4)	n.d.	n.d.	185
	α	D-Serine	267 ± 46	127 ± 17	394 ± 49 (11)	3.7 ± 1.0 (1.6)	23 ± 5 (8.0)	138 (280)
	α	L-Serine	318 ± 72*	942 ± 89	1261 ± 114* (35)	5.4 ± 1.9 (2.3)	28 ± 7 (9.8)	138 (280)
	β	D-Isoserine^#^	tr.	57 ± 12	57 ± 12 (1.6)	14 ± 2 (2.3)	29 ± 7 (10)	138
	β	L-Isoserine^#^	tr.	57 ± 6	57 ± 6 (1.6)	15 ± 2 (6.3)	33 ± 7 (11)	138
	β	β-Alanine	1064 ± 151	658 ± 36	1721 ± 156 (48)	43 ± 6 (18)	198 ± 39 (69)	168
4	α	α-AIBA	863 ± 154	152 ± 18	1015 ± 155 (28)	n.d.	n.d.	154
	α	D-α-ABA	532 ± 81	341 ± 12	873 ± 82 (24)	1.5 ± 0.7 (0.6)	63 ± 11 (22)	154 (107)
	α	L-α-ABA	541 ± 142	403 ± 70	944 ± 159 (26)	2.9 ± 0.9 (1.2)	62 ± 10 (22)	154 (107)
	α	D-Aspartic acid	108 ± 18	193 ± 33	301 ± 37 (8.4)	n.d.	1.3 ± 0.3 (0.45)	184
	α	L-Aspartic acid	267 ± 53	780 ± 117	1048 ± 129 (29)	tr.	2.5 ± 0.7 (0.87)	184
	α	D-Threonine	tr.	tr.	tr.	n.d.	n.d.	152
	α	L-Threonine	173 ± 23	519 ± 42	691 ± 48 (19)	tr.	6.9 ± 2.6 (2.4)	152
	α	D-allo-Threonine	n.d.	10 ± 1	10 ± 1 (0.28)	n.d.	n.d.	153
	α	L-allo-Threonine	n.d.	tr.	tr.	n.d.	n.d.	153
	α	DL-α-Methylserine^#^	79 ± 6*	65 ± 5*	144 ± 8* (4.0)	n.d.	74 ± 13 (26)	152 (184)
	α	D-Homoserine^#^	7.9 ± 1.0	14 ± 2*	22 ± 2* (0.62)	n.d.	9.0 ± 1.6 (3.1)	84 (152, 266)
	α	L-Homoserine^#^	13 ± 3	34 ± 6*	47 ± 6 (1.3)	n.d.	10 ± 2 (3.5)	84 (152, 266)
	β	D-β-ABA	147 ± 17*	53 ± 3*	200 ± 17* (5.6)	19 ± 1 (8.0)	45 ± 9* (16)	140 (153, 182)
	β	L-β-ABA	146 ± 31*	48 ± 3*	193 ± 31* (5.4)	24 ± 2* (10)	41 ± 4* (14)	140 (153, 182)
	β	D-β-AIBA	150 ± 54	85 ± 4*	235 ± 54* (6.6)	n.d.	16 ± 2* (5.6)	182 (69)
	β	L-β-AIBA	131 ± 50*	74 ± 1*	204 ± 50* (5.7)	n.d.	15 ± 2*(5.2)	182 (69)
	β	DL-β-Homoserine^#^	12 ± 2*	8.3 ± 1.9*	20 ± 3* (0.56)	n.d.	20 ± 4 (6.9)	180 (294)
	β	DL-3-Amino-2-(hydroxy-methyl) propanoic acid^#^	33 ± 10	32 ± 8	65 ± 13 (1.8)	9.2 ± 1.8 (3.9)	114 ± 28 (40)	180 (197)
	β	DL-Isothreonine^#^	15 ± 1*	76 ± 2*	92 ± 3* (2.6)	16 ± 2 (6.8)	61 ± 15 (21)	152 (294)
	β	D-allo-Isothreonine^#^	tr.	59 ± 12*	59 ± 12* (1.7)	17 ± 4 (7.2)	42 ± 12 (15)	294 (266)
	β	L-allo-Isothreonine^#^	tr.	50 ± 5*	50 ± 5* (1.4)	21 ± 2 (8.9)	47 ± 14 (16)	294 (266)
	γ	DL-4-A-2-HBA^#^	28 ± 1	52 ± 12	80 ± 12 (2.2)	2.8 ± 0.7 (1.2)	21 ± 4 (7.3)	153
	γ	D-4-A-3-HBA^#^	21 ± 11*	11 ± 5*	32 ± 12* (0.90)	n.d.	n.d.	152
	γ	L-4-A-3-HBA^#^	12 ± 4	7.4 ± 3.1	19 ± 5 (0.53)	n.d.	n.d.	152
	γ	γ-ABA	1244 ± 242	638 ± 35	1882 ± 245 (53)	n.d.	tr.	182
5	α	D-Valine	58 ± 1	30 ± 1	87 ± 1 (2.4)	n.d.	n.d.	55
	α	L-Valine	129 ± 15*	419 ± 62*	548 ± 64* (15)	n.d.	n.d.	55
	α	D-Norvaline	75 ± 19	55 ± 5	129 ± 20 (3.6)	n.d.	n.d.	168
	α	L-Norvaline	67 ± 12	52 ± 7	119 ± 14 (3.3)	n.d.	n.d.	168
	α	DL-Isovaline	1418 ± 247	250 ± 20	1668 ± 248 (47)	n.d.	n.d.	168
	α	D-Glutamic acid	172 ± 27	142 ± 25	314 ± 37 (8.8)	n.d.	n.d.	180
	α	L-Glutamic acid	426 ± 85	788 ± 115	1214 ± 143 (34)	tr.	tr.	180
	β	D-β-(Aminomethyl)-succinic acid^#^	30 ± 5	16 ± 4	46 ± 7 (1.3)	n.d.	1.9 ± 0.6* (0.66)	226
	β	L-β-(Aminomethyl)-succinic acid^#^	27 ± 4*	17 ± 2*	44 ± 4* (1.2)	n.d.	2.4 ± 0.7* (0.83)	226
6	α	D-α-Aminoadipic acid	115 ± 24	27 ± 4*	142 ± 25* (4.0)	n.d.	n.d.	194 (212)
	α	L-α-Aminoadipic acid	118 ± 35*	66 ± 9	184 ± 36* (5.2)	n.d.	n.d.	194 (212)
	α	D-Leucine	17 ± 1*	63 ± 1*	80 ± 1* (2.6)	n.d.	n.d.	140 (182)
	α	L-Leucine	170 ± 34	888 ± 13	1058 ± 37 (30)	n.d.	tr.	140 (182)
	α	D-Isoleucine	101 ± 9	172 ± 1	273 ± 9 (7.7)	n.d.	n.d.	182
	α	L-Isoleucine	251 ± 60	696 ± 54	947 ± 81 (27)	n.d.	tr.	182
		Total	11636 ± 506	12500 ± 249	24136 ± 564	460 ± 28	1576 ± 82	
		HAA total***	221 ± 17	523 ± 26	744 ± 31	95 ± 6	460 ± 41	

n.d.: not detected; tr.: trace amount.

^#^Firstly identified in the Murchison meteorite.

*The chromatographic peak overlapped with other peak(s) including its enantiomer.

**The *m/z* was used for the quantification of amino acids. The *m/z* in parenthesis was secondarily used.

***The total concentration of newly identified hydroxy amino acids.

**Figure 1:**
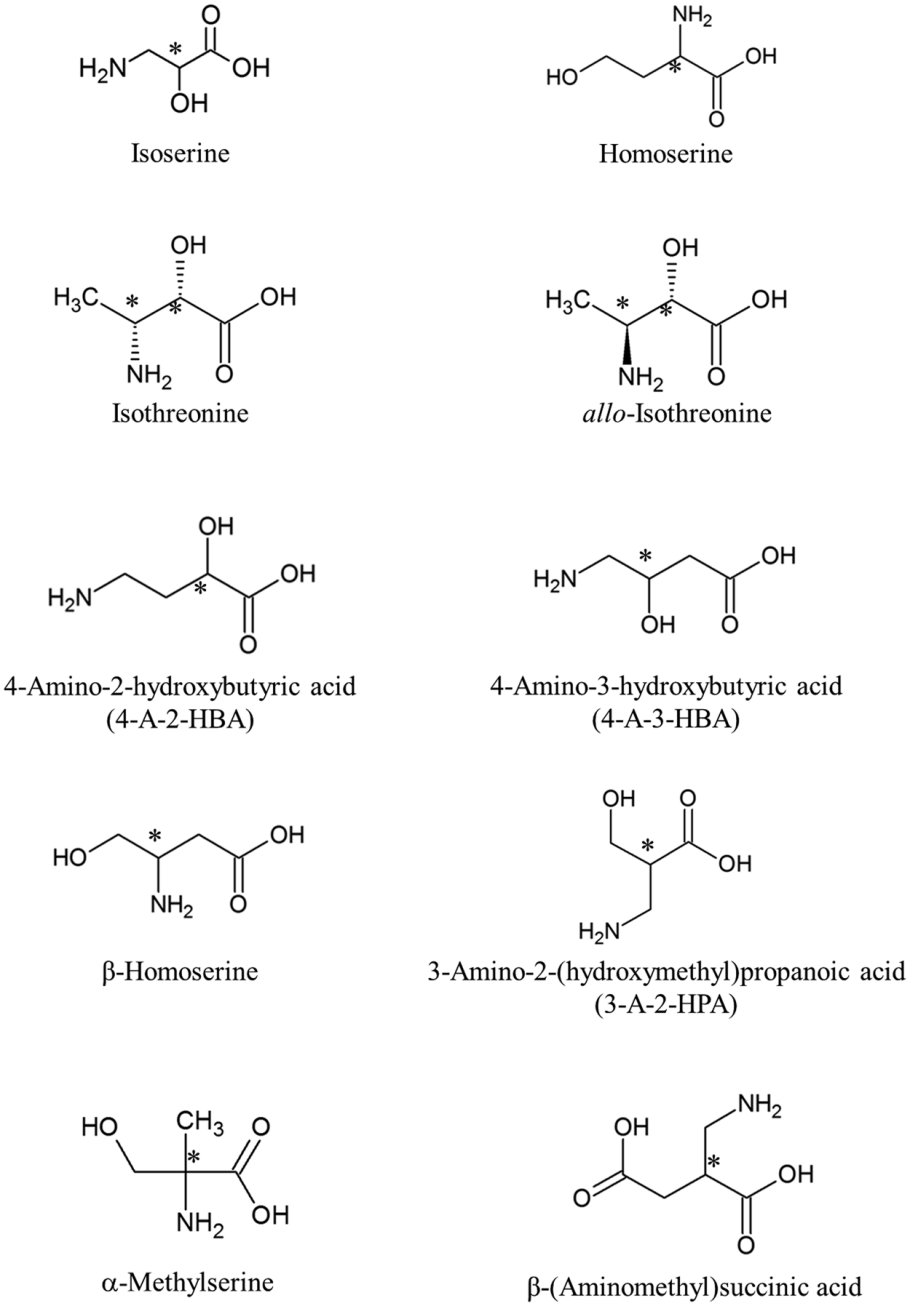
Chemical structures of amino acids newly identified in the Murchison meteorite. *Asymmetric carbon.

Nine hydroxy amino acids (HAAs) including isoserine, homoserine, β-homoserine, α-methylserine, 4-amino-2-hydroxybutanoic acid (4-A-2-HBA), 4-amino-3-hydroxybutanoic acid (4-A-3-HBA), isothreonine, *allo*-isothreonine and 3-amino-2-(hydroxymethyl)-propanoic acid (3-A-2-HPA), as shown in Fig. 1 for their structures and the SI for their mass spectra, have been identified in the Murchison meteorite for the first time. Isoserine is a C_3_ β-amino acid, the structural isomer of serine. Even though it was identified from Antarctic CR meteorites^16^, this study revealed the first occurrence of isoserine in the Murchison meteorite. Eight additional HAAs are C_4_ structural isomers including two α-, four β- and two γ-amino acids. Any organic compounds bearing –OH and –NH_2_ at the same carbon are unstable and are unlikely to occur in the natural environment. Furthermore, four HAAs (one C_4_ and three C_5_) were found based on their mass spectra (see SI), but their structures could not be assigned due to the lack of appropriate standards. The newly discovered HAAs were present as near-racemic mixtures except for homoserine, if their DL forms can be separated by chromatography (see SI). In addition, a new C_5_ dicarboxy amino acid, β-amino acid β-(aminomethyl)succinic acid, was identified in meteorites, although one C_6_ β-amino dicarboxyl acid (3-aminoadipic acid) was reported in Murchison^17^.

The total concentration of the new HAAs ranged from ~20 to ~140 ppb (Table 1). Although the direct HCl hydrolysis of the extract residue is not commonly performed, the amino acid occurrence was different between the water extract and the extract residue. The HAAs were generally present in higher concentrations in the extract residue relative to the water extract. In particular, isoserine and *allo*-isothreonine were detected in trace amounts in the water extract (Table 1). In contrast, α-Methyl amino acids such as AIBA and isovaline were six times more abundant in the water extract relative to the extract residue.

### Amino acids produced from aldehydes and ammonia in aqueous solution

As a complement to the analysis of amino acids in the Murchison meteorite, heating experiments were performed using a mixture of formaldehyde, acetaldehyde and ammonia in aqueous solution in the presence or absence of glycolaldehyde at 60 °C for 6 days (pH = 11.5 for the starting solution), to simulate possible chemical reaction during aqueous alteration of the meteorite parent body. Aldehydes such as formaldehyde and acetaldehyde are abundant in molecular clouds (e.g. ref. 18) and are probably the predominant carbon sources to produce meteoritic organic compounds such as sugars by the formose reaction^19, 20^. Glycolaldehyde was also used to evaluate the first reaction step, as glycolaldehyde can condense from two formaldehydes by the formose reaction. Ammonia has been detected in high concentrations in the Murchison meteorite^21^ and is the principal nitrogen source for organic chemistry in meteorites.

Various amino acids including glycine and HAAs were synthesized from aldehydes and ammonia (Table 1). While glycine (C_2_) was the most abundant amino acid, followed by alanine or β-alanine (C_3_), α-methyl alkylated (no hydroxy) amino acids such as AIB and isovaline were not formed in the experiments. The number of amino acid species produced and their concentrations increased in the glycolaldehyde-present vs. glycolaldehyde-absent experiments. In the presence of glycolaldehyde, larger (≥C_4_) amino acids were found in more diverse structures. In particular, more C_4_ HAAs and at higher concentrations, were produced when the starting material included glycolaldehyde (Table 1).

## Discussion

### A new family of hydroxy amino acids in the Murchison meteorite

Previously only three HAAs (serine, threonine and *allo*-threonine) have been identified in the Murchison meteorite^4^. These are all α-amino acids, in which serine and threonine are proteinogenic amino acids and *allo*-threonine is a diastereoisomer of threonine, which have often been present as an L-rich signature. In particular, the presence of L-threonine detection (~700 ppb concentration) with only trace amounts of D-threonine (Table 1 and SI) is suggestive of terrestrial contamination. Non-proteinogenic HAAs such as isoserine and homoserine have previously been identified in a few Antarctic CR meteorites^16^, but to the best of our knowledge, they have not previously been reported from the Murchison meteorite. In this study, nine new C_3_ and C_4_ HAAs were discovered in the Murchison meteorite, comprising a new family of meteoritic amino acids. Three C_4_ additional HAAs (α-methylisoserine, N-methylserine and N-methylisoserine) were not positively identified currently due to the lack of the appropriate standard or the low intensity of mass peaks (see SI). The finding of most structural isomers of C_3_ and C_4_ HAAs expands the structural diversity of soluble meteoritic organic compounds, particularly for small amino acids (≤C_4_). The three C_5_ HAAs were identified by mass spectra that are characteristic of their structures (see SI), although these identifications are somewhat tentative, as the complete structures were not determined due to the necessary standards being unavailable.

It is notable that these HAAs have never been found before in the Murchison meteorite, despite numerous rigorous surveys of amino acids over almost 50 years. Although the C_4_ HAAs were present in concentrations less than the major amino acids such as alanine and aminobutyric acids, the concentrations were comparable to those of the C_5_ amino acids (Table 1). We suggest that these HAAs have not been identified before as they are non-proteinogenic amino acids and it is relatively difficult to prepare their standards. Moreover, the hydroxyl amino acids were generally present in higher concentrations in the extract residue vs. the water extract. For example, isoserine and *allo*-isothreonine were only detected in trace amounts in the water extract, but were present in significant concentrations in the extract residue (Table 1), possibly due to the hydroxyl group being intimately adsorbed or chemically bound with clay minerals, and hence being poorly extracted by water. The direct HCl treatment of the extract residue may have yielded the HAAs due to the dissolution of the clay minerals and/or due to protonation of the clay mineral surfaces. The detection of isoserine and homoserine in the water extract of CR meteorites^14^, which are less aqueously altered than CM chondrites^1^, may be due to the presence of less abundant clay minerals in the CR chondrites.

The chirality of HAAs was very different between proteinogenic (serine and threonine) and the newly identified non-proteinogenic HAAs in this study. Serine and threonine showed strong L-enrichment, while non-proteinogenic HAAs were present as nearly racemic mixtures. One exception (homoserine) apparently showed the L-enrichment when considering the fragment ion with *m/z* 84. However, when considering the *m/z* 152 and *m/z* 267 fragment ions for DL resolution, the chromatograms showed D-enrichment (see SI). Because the amino acid fraction was a complex mixture, the L-preference of homoserine in this study is not conclusive. Possible racemization during the analytical procedures was minimized, demonstrated by D-threonine being detected only in trace amounts. The small amount of D-allo-threonine could be formed from L-threonine, since L-threonine is known to be epimerized to the D-allo-threonine e.g. ref. 22. The racemization experiment of L-HAA standards also showed that the racemization was minimal (up to 1.6%) during the analytical procedure (see SI). Therefore, the occurrence of racemic HAAs implies non-asymmetric synthesis in the formation pathway(s).

### Comparison of amino acid occurrence between the Murchison meteorite and experiments

The relative abundance of amino acids was normalized to glycine abundance, the simplest and most abundant amino acid in the Murchison meteorite as well as the experiments (Table 1). Although the C_3_ α-amino acids (e.g. alanine) were abundant in both the Murchison meteorite and the experiments, the experimental products were relatively depleted in larger (≥C_4_) alkylated α-amino acids vs. the Murchison extracts. In particular, α-methyl alkylated (OH-free) amino acids such as AIBA and isovaline were present only in the Murchison meteorite, and were not detected in the experimental products. The larger alkylated and α-methyl amino acids were likely synthesized using the corresponding aldehydes or ketones by the Strecker reaction. However, the experimental result clearly indicates that the α-amino acids can be synthesized from aldehydes and ammonia, and that this reaction does not need cyanide, which is required for the Strecker reaction to proceed. Therefore, a certain fraction of the meteoritic α-amino acids appear to be produced from aldehydes under an NH_3_-rich condition.

**Figure 2:**
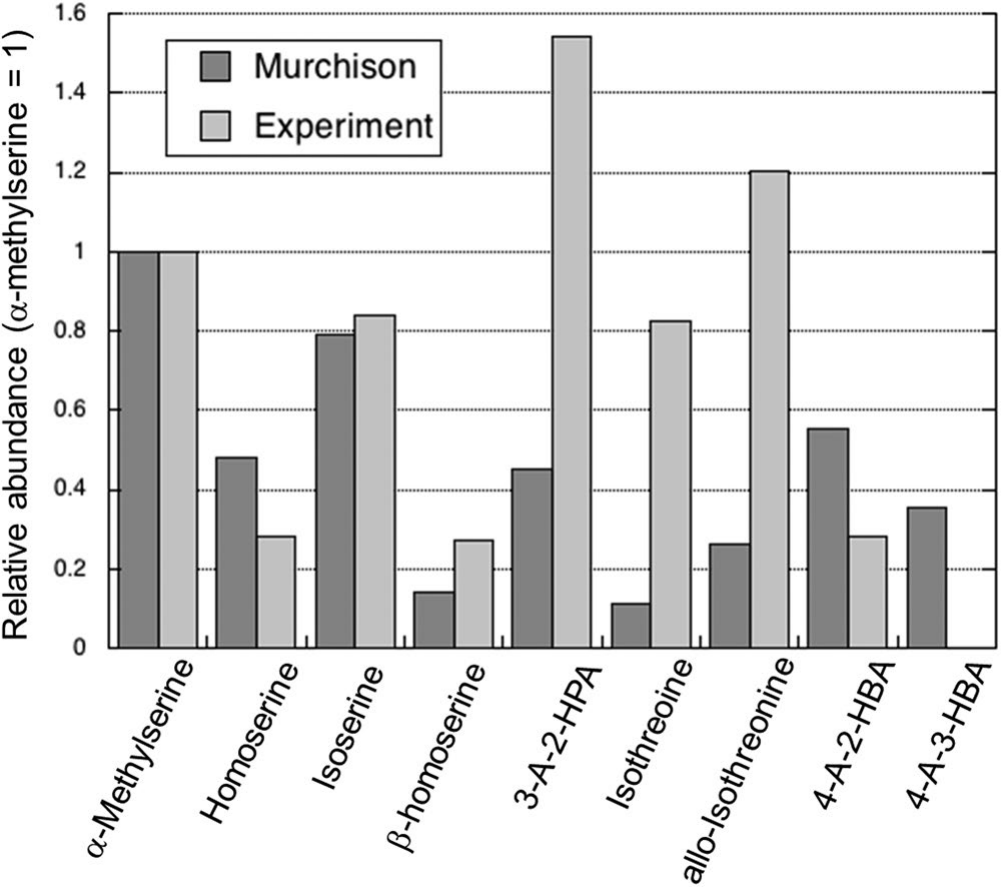
Relative abundance of the newly identified hydroxy amino acids between the Murchison meteorite and the experiment.

The HAAs newly identified in the Murchison meteorite were effectively synthesized by the experiments, except for 4-A-3-HBA. In Fig. 2, the relative abundance of HAAs was compared between the Murchison meteorite and the experimental product in the presence of glycolaldehyde (α-methylserine = 1), which shows a similarity between the serine-derivatives (isoserine, homoserine and β-homoserine). However, other β-HAAs have larger abundances in the experiment, while γ-HAAs have larger abundances in the Murchison meteorite. In particular, no 4-A-3-HBA was produced in the experiment, which probably reflects the extent of carbon elongation depending upon the starting materials and/or regulation by minerals in the meteorite.

In contrast, threonine and its epimer allo-threonine were not detected in the experimental products except for small amount of L-threonine which is likely a contaminant because of its small occurrence in the procedural blank. D-threonine and L-allo-threonine were detected only in trace amounts in the Murchison meteorite, and a small amount of D-allo-threonine could be the product of the epimerization of the large amount of L-threonine present. Even though the L-threonine enrichment has been proposed to be indigenous to the meteorites^13^, the occurrence of threonine and allo-threonine was consistent between the meteorite and the experiment in this study if L-threonine is considered to be a contaminant. In addition to HAAs, of the β-amino acids identified, β-alanine and β-(2-)amino butanoic acid were relatively abundant in both the Murchison meteorite and the experiments, suggesting a common formation pathway other than the Strecker reaction. The newly identified dicarboxy-β-amino acid, i.e. 2-(aminomethyl)succinic acid, also suggests a similar mechanism for the production of β-amino acids from aldehydes and ammonia (see below).

### Formation pathways of meteoritic amino acids

The Strecker reaction has long been considered as the principal mechanism to produce meteoritic α-amino acids^4, 11^. Hydroxy α-amino acids such as serine, threonine, homoserine and α-methylserine can be produced by electric discharge using CH_4_, NH_3_, H_2_ and H_2_O^23^. These are all hydroxy α-amino acids, suggesting the Strecker reaction as a possible formation mechanism. The occurrence of β and γ amino acids suggests the possibility of other mechanisms to produce meteoritic amino acids. In particular, our experimental results suggest that the meteoritic HAAs could be produced from aldehydes and ammonia through a formose reaction and aldol condensation. The total concentration of newly identified HAAs was 744 ppb relative to the bulk meteorite and approximately 40 ppm relative to the total C, since the bulk C concentration of the Murchison meteorite ranged from 1.6 to 2.5 wt% (e.g. ref. 24). In contrast, the concentration of the HAAs in the experiment was 460 ppm relative to the total C of the starting material. Therefore, the concentration of HAAs in the Murchison meteorite is comparable (about 10%) relative to the concentration of the HAAs in the experiment. Hence, it is quantitatively assumed that the meteoritic HAAs could be produced through a synthesis using aldehydes and ammonia.

**Figure 3:**
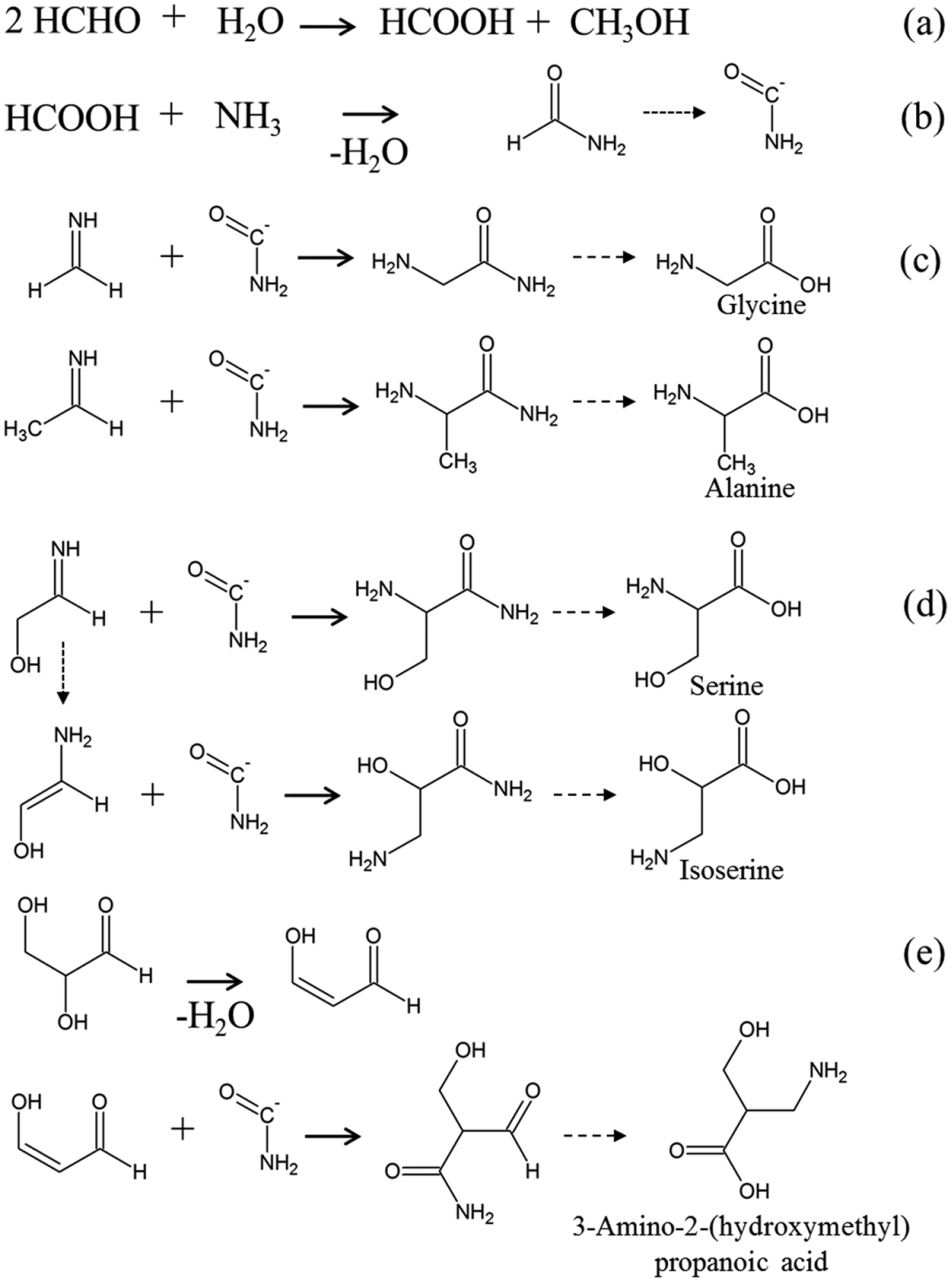
Reaction scheme producing amino acids (glycine, alanine and hydroxy amino acids) from aldehydes and ammonia.

A possible formation pathway for HAAs is proposed in Fig. 3. Initially formaldehyde disproportionates under alkaline conditions, known as the Cannizzaro reaction, to produce formic acid (a). The formic acid produces formamide by reaction with ammonia, followed by production of the carbomyl anion (b). The carbomyl anion then reacts with formaldehyde and acetaldehyde, producing glycine and alanine respectively after hydrolysis (c). Similarly, serine and isoserine can be produced by the reaction of the carbomyl anion with glycolaldehyde (d). Addition of the carbomyl anion to C_3_ keto-enol components, produced by aldol condensation, then yields various C_4_ HAAs (e). These processes may have multiple routes through rearrangement and/or cyclisation. Furthermore, 2-(aminomethyl)succinic acid can be produced from succinimide, which was identified in the Murchison meteorite^7^, resulting from the formose reaction with ammonia (see SI). It is known that the formation of glycolaldehyde accelerates further condensation of aldehydes to lengthen the carbon chains^25^. Therefore, the first step of formose reaction using two formaldehydes is the rate-determining step for production of the larger amino acids, as shown in the glycolaldehyde-present experiment. Hence, formation mechanism(s) other than the Strecker reaction are implied for β, γ and δ meteoritic amino acids, and are also possible for some α-amino acids.

The proposed formation pathways are also consistent with the occurrence of other soluble organic compounds, including the C_3_-C_6_ sugar-related compounds^19^ and alkylated (up to C_26_) pyridines^26^ in the Murchison meteorite. The sugar-related compounds can be produced from formaldehyde through the formose reaction under alkaline conditions. Moreover, the alkylated pyridines can be synthesized by the aldol condensation and imine formation from aldehydes under alkaline conditions with ammonia. Furthermore, it is suggested that chondritic insoluble organic matter was produced through polymerization of formaldehyde followed by condensation upon heating probably on the parent body^27, 28^. The parent body of many carbonaceous chondrites, including the Murchison meteorite, suffered aqueous alteration at 20–100 °C^1, 29^. This aqueous reaction can produce clay minerals, such as serpentine, by alteration of anhydrous silicates (e.g. olivine and pyroxene), which consumes protons and results in alkaline conditions. Therefore, the minerals present in meteorites may also have important roles as catalysts to control organic reactions.

In prebiotic chemistry in the primitive asteroid, simple aldehydes such as formaldehyde and acetaldehyde could be the principal carbon sources to synthesize various organic compounds through their polymerization in the presence of NH_3_ under alkaline conditions. Hence, further investigation is needed to reveal possible organic-mineral interactions for the better understanding of chemical evolution in the solar system.

## Methods

### Amino acid analysis of Murchison

The detailed analytical procedure is described in the Supplementary Information (SI). Briefly, powdered Murchison meteorite (425 mg) was extracted with water at 100 °C for 20 h. The supernatant (water extract) and the extract residue were subjected to acid hydrolysis with 3 M and 6 M HCl, respectively, at 105 °C for 20 h. After removing ether-soluble organic components, the resulting solution was desalted using an ion exchange column. The purified amino acids were converted to trifluoroacetyl-amino acid-isopropyl ester derivatives to be analyzed by gas chromatography/mass spectrometry with a Chirasil-L-Val capillary column. The amino acids were identified based on their retention times and the mass spectra of standard amino acids, and quantified by comparison of the peak area using characteristic fragment ions of each amino acid. The amino acid concentration was calculated vs. the bulk meteorite (ppb). A pre-heated sea sand was used for the procedural blank, and less than 1.3% of L-serine, glycine, L-leucine, L-alanine, L-threonine, L-glutamic acid and L-aspartic acid were detected relative to the corresponding amino acids in the Murchison meteorite (see SI in detail). The possible racemization was evaluated using the L-hydroxy amino acid standards, suggesting a small racemization (up to 1.6%) during the analytical procedure (see SI in more detail).

### Experimental amino acid synthesis

An aqueous solution (300 μL) containing H_2_O/ammonia/formaldehyde/acetaldehyde (1000/100/10/1 molar ratio) or H_2_O/ammonia/formaldehyde/acetaldehyde/glycolaldehyde (1000/100/10/1/1 molar ratio) was heated at 60 °C for 6 days in a N_2_-purged glass ampoule. The molar ratio of H_2_O/ammonia/formaldehyde was adapted from the observed interstellar and cometary ice compositions^18^. The reaction products were hydrolyzed followed by derivatization, and analyzed using the same procedure as described above. The amino acid concentration was calculated relative to the total initial carbon concentration of aldehydes (ppm).

## Electronic supplementary material

Supplementary Info
