# Supplementary material for: A new family of extraterrestrial amino acids in the Murchison meteorite
Source: Sci Rep. 2017 Apr 4;7:636. doi: 10.1038/s41598-017-00693-9 (PMC5428853; doi:10.1038/s41598-017-00693-9)

A new family of extraterrestrial amino acids in the Murchison meteorite
by Koga & Naraoka

Supplementary Information

1. Amino acid analysis of the Murchison meteorite

The Murchison meteorite used in this study was obtained from the Field Museum, Chicago and stored in a desiccator with silica gel at room temperature. The interior fragments (425 mg) were powdered using an agate mortar and pestle in a clean bench. The powdered sample was extracted with 2 mL of distilled and deionized H_2O (18.2 MΩ Milli-Q water, Merck Millipore) at 100°C for 20 h in a N_2-purged glass ampoule. After centrifugation, the supernatant was removed and the residue was extracted with 1 mL of H_2O by sonication followed by centrifugation. The combined supernatant and the extract residue were subjected to acid hydrolysis with 3M and 6M HCl, respectively, at 105°C for 20 h in a N_2-purged glass ampoule. The acid-hydrolyzed solutions were extracted with diethyl ether (1mL) to remove ether-soluble organic compounds, and the resulting acid solutions were evaporated to dryness under vacuum. After redissolving the extract in 1M HCl (~0.3mL), the extracts were desalted using an ion exchange column (DOWEX 50X-8, 50–100 mesh, hydrogen form). The amino acid fraction was eluted with 3 mL of ammonium hydroxide, followed by drying under vacuum. The amino acids were reacted with 0.6 mL of isopropanol (iPrOH)/HCl (1.25M, Sigma-Aldrich) at 70°C for 3 h a N_2-purged glass ampoule. After removing the solvent under a stream of N_2 gas, the amino acid-OiPr derivatives were further reacted with 600 μL of trifluoroacetic anhydrite (TFAA, Sigma-Aldrich) overnight at room temperature in a N_2-purged glass ampoule. The resultant TFA-amino acid-OiPr derivatives were extracted with 2mL of dichloromethane and concentrated to 50 μL under a stream of N_2 gas. All glassware were baked at 500°C for 3 hours prior to use to remove possible organic contaminants. The procedural blank was analyzed using a pre-heated (500°C for 3 h) sea sand by the same method.

The amino acid derivatives were analyzed using a gas chromatograph-mass spectrometer (Trace GC coupled with Polaris Q MS, Thermo Scientific) with a CP ChiraSil-L-Val capillary column (25 m x 0.25 mm i.d., 0.12 μm film thickness, Agilent) in splitless mode at an injector temperature of 200°C. The GC column was programmed from 40°C (2 min) to 80°C at 20°C min^{-1} then to 180°C at 4°C min^{-1}

with He as a carrier gas (1 mLmin^{-1}). The amino acids were identified based on their retention times and the mass spectra of the standard amino acids.

The concentrations of amino acids were determined by comparison with the calibration curves of standards using the peak area of a specific fragment ion. The average and standard deviation (2*s*) were calculated by two or three injections relative to the bulk meteorite in ppb for Murchison and relative to the total carbon contents of aldehydes in ppm for the experiments (Table 1). The procedural blank gave L-serine (16.2±2.0 ppb), glycine (13.2±1.3 ppb), L-leucine (9.3±1.1 ppb), L-alanine (4.2±0.3ppb), L-threonine (3.2±0.4 ppb), L-glutamic acid (2.6±0.2 ppb) and L-aspartic acid (1.5±0.2 ppb), which were less than 1.3% (L-serine most abundant) relative to the corresponding L-amino acids detected in the Murchison meteorite.

In order to evaluate the recemization of hydroxy amino acids during the analytical procedure, the heating experiment was performed using L-hydroxy amino acid standards including L-homoserine, L-β-homoserine and L-4-amino-2-butanoic acid with 6M HCl at 105 °C for 20 h in the presence of serpentine (antigorite) in a N_2-purged glass ampoule, which was the similar condition as the actual analytical procedure. As a result, no recemization was observed for L-β-homoserine and L-4-amino-2-butanoic acid, although the former tiny D-peak may not be discriminated from the later large L-peak due to their close retention times. A little recemization (1.6%) of L-homoserine was observed, suggesting the minimal racemization during the analytical procedure.

The identification and quantification of each amino acid are explained as follows. If only the L-enantiomer is available as a standard, the D-enantiomer can be identified using the retention times and the mass spectra of the experimental products of the amino acid synthesis as well as the racemization experiment. For the racemic standards of hydroxy amino acids, we postulate that the D-enantiomer elutes faster that the corresponding L-enantiomer since most D amino acids (especially for all proteinogenic amino acids) elute faster than their corresponding L-enantiomers using the Chirasil-L-Val column. The appropriate *m/z* was selected to quantify due to overlapping peaks resulting from complex mixtures on the chromatogram.

1) Isoserine: The quantification was carried out using *m/z* 138. The DL-isoserine was resolved and mass spectra matched well between Murchison and standard.
2) Homoserine: Apparent L-enrichment was observed in Murchison using a fragment ion of *m/z* 84. However, if other *m/z* 152 and *m/z* 267 were used for the DL

resolution, the chromatograms showed D-enrichment. The potential overlapping peak(s) could not be excluded in the retention time of this range. The L-preference of homoserine in this study is not conclusive.

3) β-Homoserine: The DL-β-homoserine was not resolved well and only L-β-homoserine was available as a standard. The base peak of m/z 180 did not show good shape due to the relatively large background from other peaks. The quantification was carried out using *m/z* 294 as a DL-mixture.

4) 3-Amino-2-(hydroxymethyl)propanoic acid (3-A-2-HPA): The racemic standard of 3-A-2-HPA showed a single peak. The background of *m/z* 180 (base peak) was relatively high in this retention time in Murchison. The quantification was carried out using *m/z* 197 as a DL-mixture..

5) 4-Amino-2-hydroxybutyric acid (4-A-2-HBA): The DL-4-A-2-HBA was not resolved well and only L-4-A-2-HBA was available as a standard. The base peak of *m/z* 153 was used for quantification.

6) 4-Amino-3-hydroxybutyric acid (4-A-3-HBA): The racemic standard of DL-4-A-3-HBA was poorly resolved with apparent slightly L-enrichment. However, it was possible to quantify each enantiomer of 4-A-3-HBA using the base peak of *m/z* 152 with a relatively large error.

7) α-Methylserine: The racemic standard of α-methylserine showed a single peak. The background of *m/z* 152 (base peak) was relatively high in this retention time in Murchison due to probable overlapping with other peaks. The second peak of *m/z* 184 was used for quantification.

8) Isothreonine: The racemic standard of isothreonine was poorly resolved with apparent L-enrichment. The relatively large peak having *m/z* 152 (base peak) and *m/z* 266 appeared in earlier retention time of Murchison, preventing the quantification. We adapted *m/z* 294, which is relatively small but independent of the background, to quantify as a DL mixture.

9) *allo*-Isothreonine: The racemic standard of *allo*-isothreonine contained small impurity of DL-threonine and DL-allo-threonine, because this standard was synthesized from DL-threonine. The standard peak of D-*allo*-isothreonine overlapped with that of L-allo-threonine as indicated by the shaded area. Although the DL-*allo*-isothreonine was resolved well, D-*allo*-isothreonine is potentially overlapped with L-allo-threonine. In the Murchison meteorite, little L-allo-threonine was detected. The base peaks (*m/z* 140 and *m/z* 152) of the *allo*-isothreonine were overlapped with relatively high backgrounds in Murchison

because a large peak between D- and L- *allo*-isothreonine had peaks of *m/z* 140 and *m/z* 152. Therefore, we used *m/z* 266 for quantification.

10) β-(Aminomethyl)succinic acid: The racemic standard of β-(Aminomethyl)-succinic acid was poorly resolved with apparent L-enrichment. However, it was possible to quantify each enantiomer of β-(Aminomethyl)succinic acid using the base peak of *m/z* 226.

2. Experimental amino acid synthesis

Aqueous solutions containing H_2O/ammonia/formaldehyde/acetaldehyde (1000/100/10/1 molar ratio) or H_2O/ammonia/formaldehyde/acetaldehyde/glycolaldehyde (1000/100/10/1/1 molar ratio) were prepared using 16% (w/v) formaldehyde solution (methanol-free, Thermo Scientific), 99% acetaldehyde (Wako) and 10% ammonia solution (Wako) with or without glycolaldehyde dimer (Sigma-Aldrich). The H_2O used was ultrapure water prepared by Milli-Q water (18.2 MΩ, Merck Millipore). The pH of the starting solution (H_2O/ammonia/formaldehyde/acetaldehyde) was 11.5, measured using a pH meter (ELP-035/HM-21P, DKK-TOA).

3. Standard preparation

Isoserine and 4-amino-3-hydroxybutanoic acid were obtained from Tokyo Chemical Industry Co., LTD. L-Homoserine, L-β-homoserine, α-methyl-DL-serine and (S)-(-)-4-amino-2-hydroxybutanoic acid were obtained from Sigma-Aldrich. N-methylserine was obtained from Watanabe Chemicals Ind., LTD. Isothreonine and allo-isothreonine were synthesized according to the method of Shimohigashi et al [1]. 3-amino-2-(hydroxy-methyl)propanoic acid and β-(aminomethyl)succinic acid were synthesized according to the method of Zilkha and Golik [2]. N-methylisoserine was synthesized from Fmoc-DL-isoserine (Watanabe Chemicals Ind., LTD) according to the method of Luo et al [3].

[1] Shimohigashi, Y., Waki, M. & Izumiya, N. Stereospecific synthesis of D-isothreoine from L-threonine. *Bull. Chem. Soc. Jpn* **52,** 949-950 (1979).

[2] Zilkha, A. & Golik, U. Syntheses of amide derivatives of DL-β-carboxy-γ-aminobutyric acid. *J. Org. Chem.* **28,** 2007-2009 (1963).

[3] Luo, Y., Evindar, G., Fishlock, D. & Lajoie, G.A. Synthesis of N-protected N-methyl serine and threonine. *Tetrahed. Lett.* **42,** 3807-3809 (2001).

4. Mass chromatograms and mass spectra of hydroxy amino acids detected in the Murchison meteorite with their procedural blank and standards. a) Murchison (the hydrolyzed extract residue), b) Blank, and c) Standard.

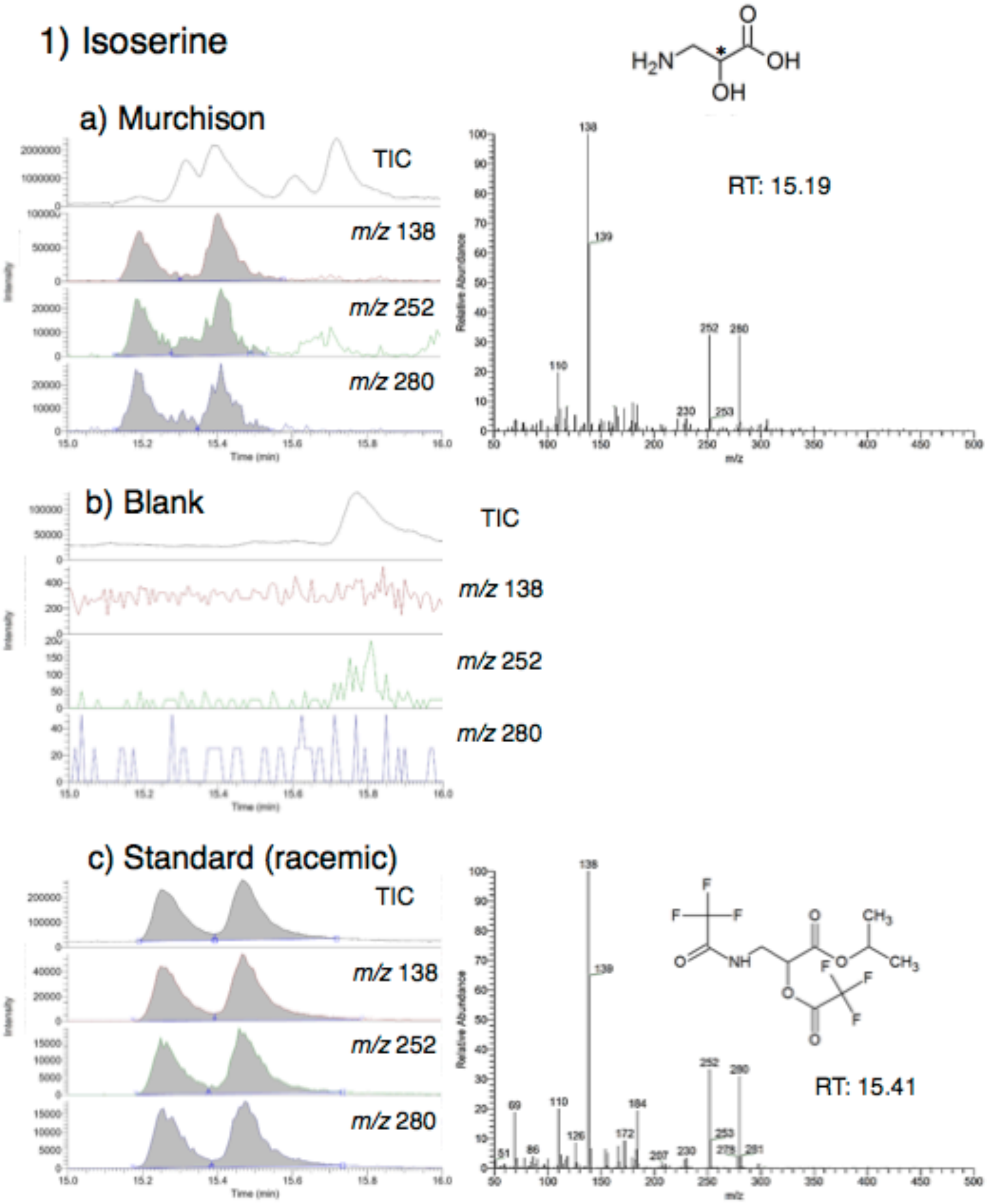

2) Homoserine

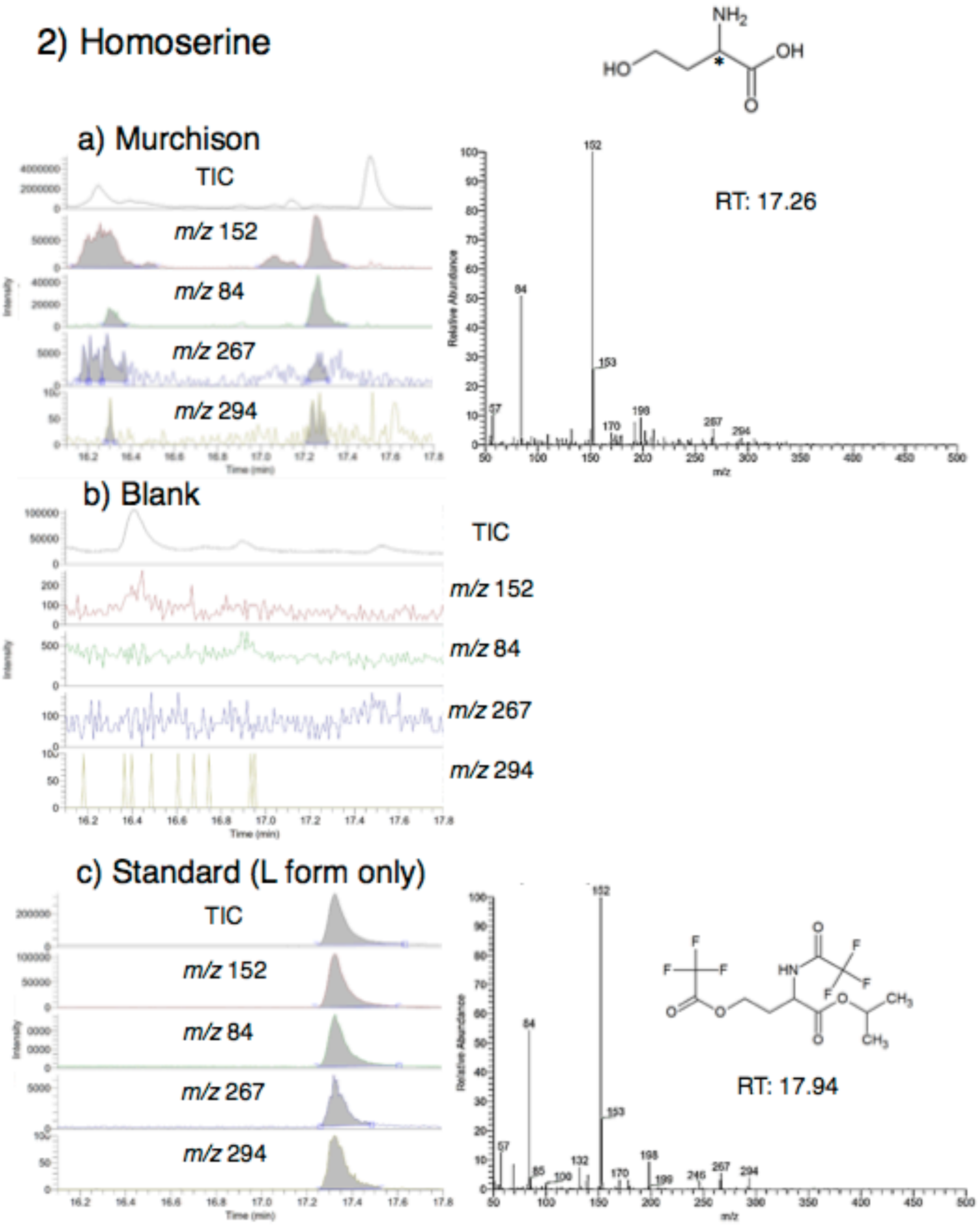

3) β-Homoserine

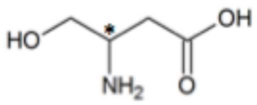

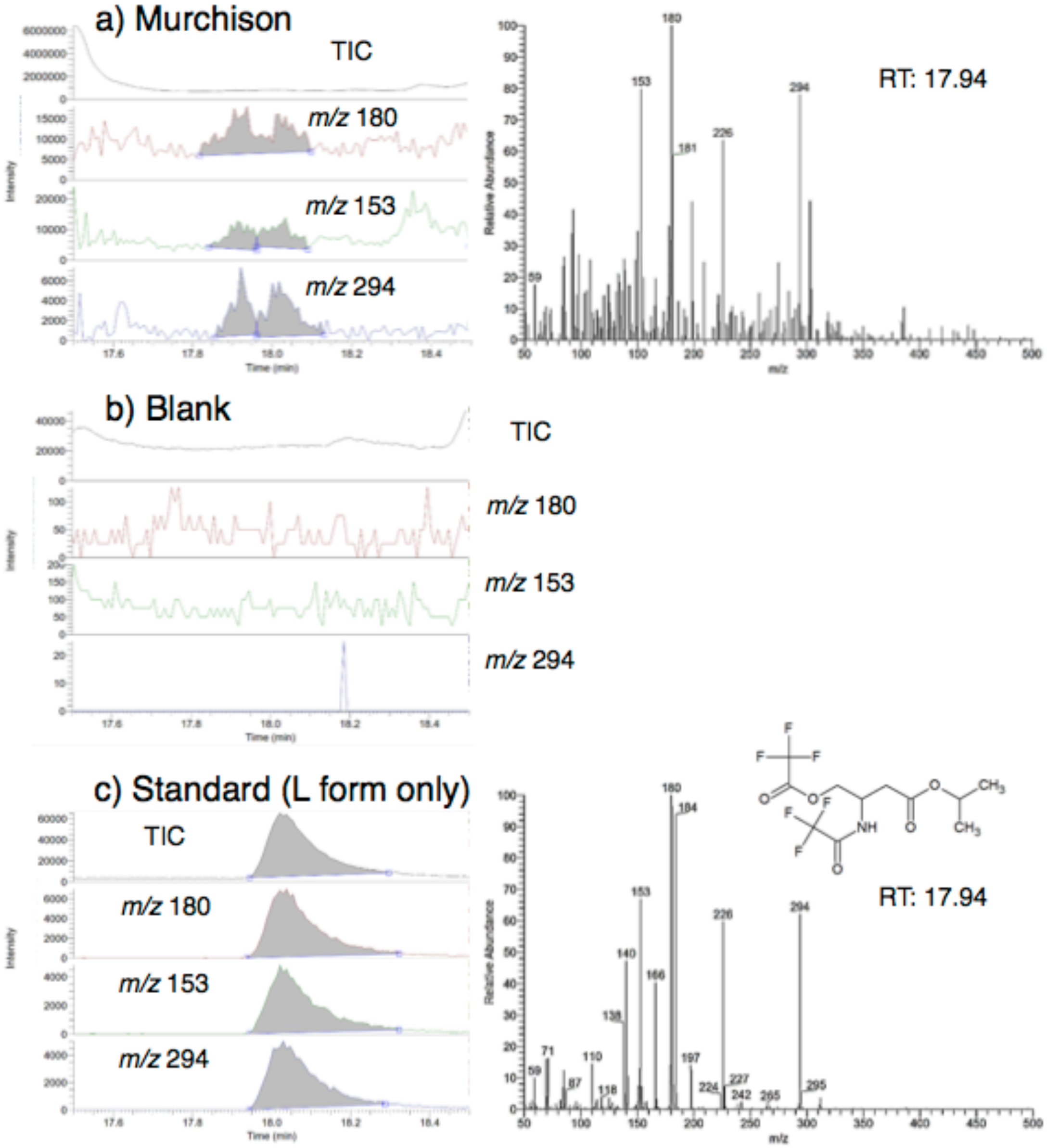

4) 3-Amino-2-(hydroxymethyl)propanoic acid

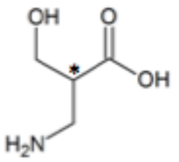

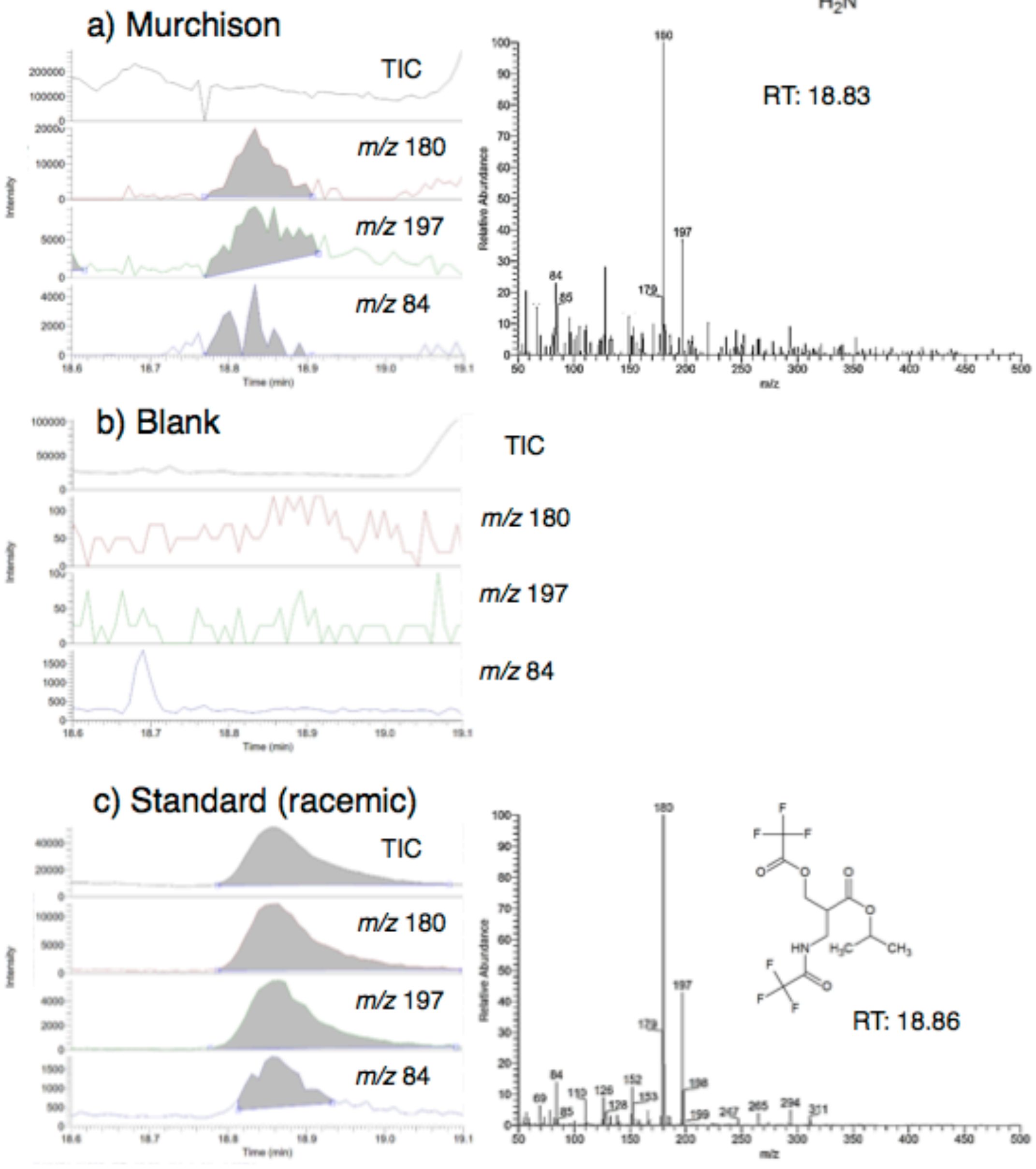

5) 4-Amino-2-hydroxybutyric acid

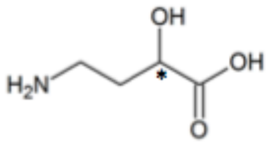

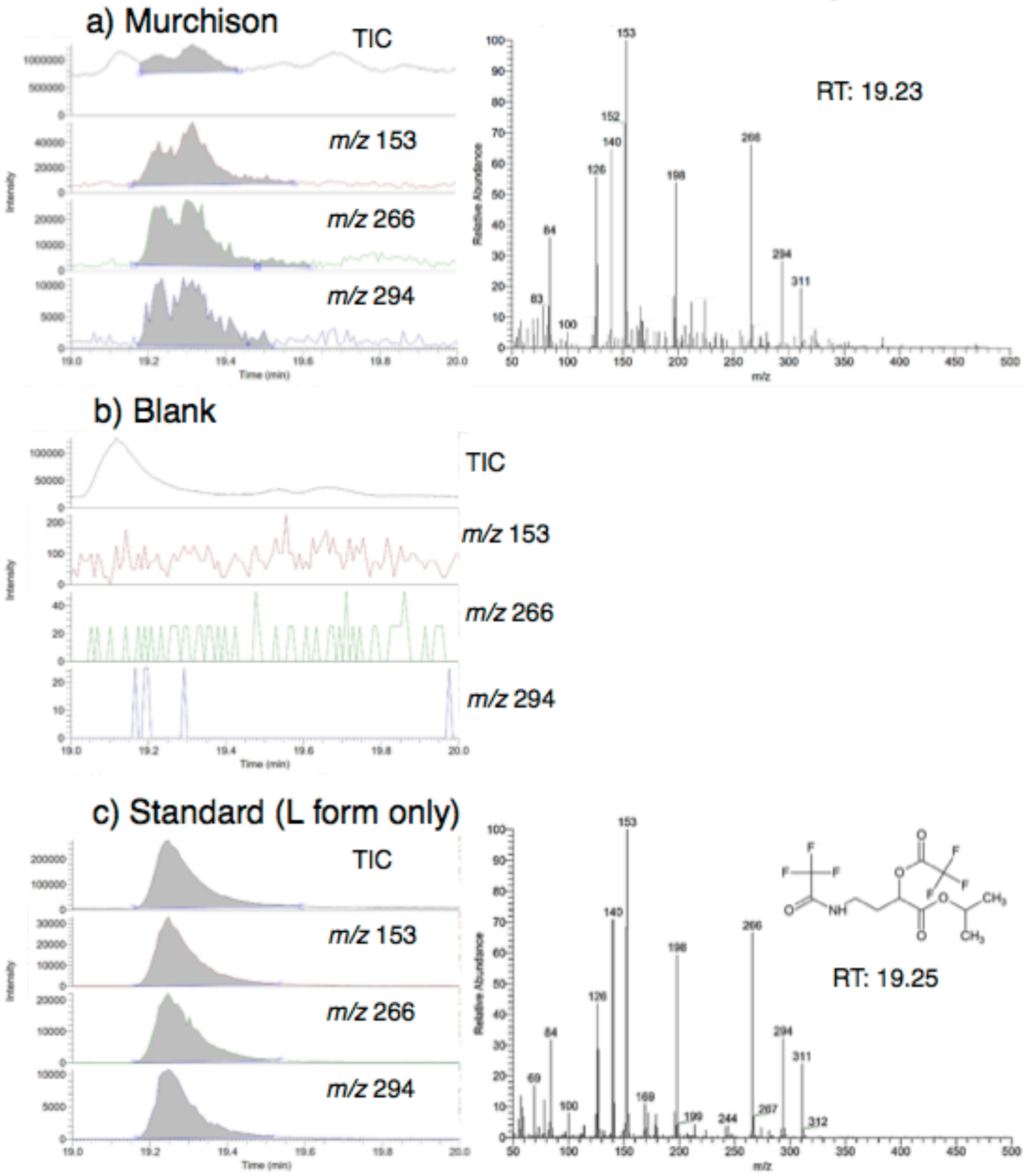

6) 4-Amino-3-hydroxybutyric acid

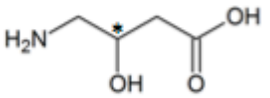

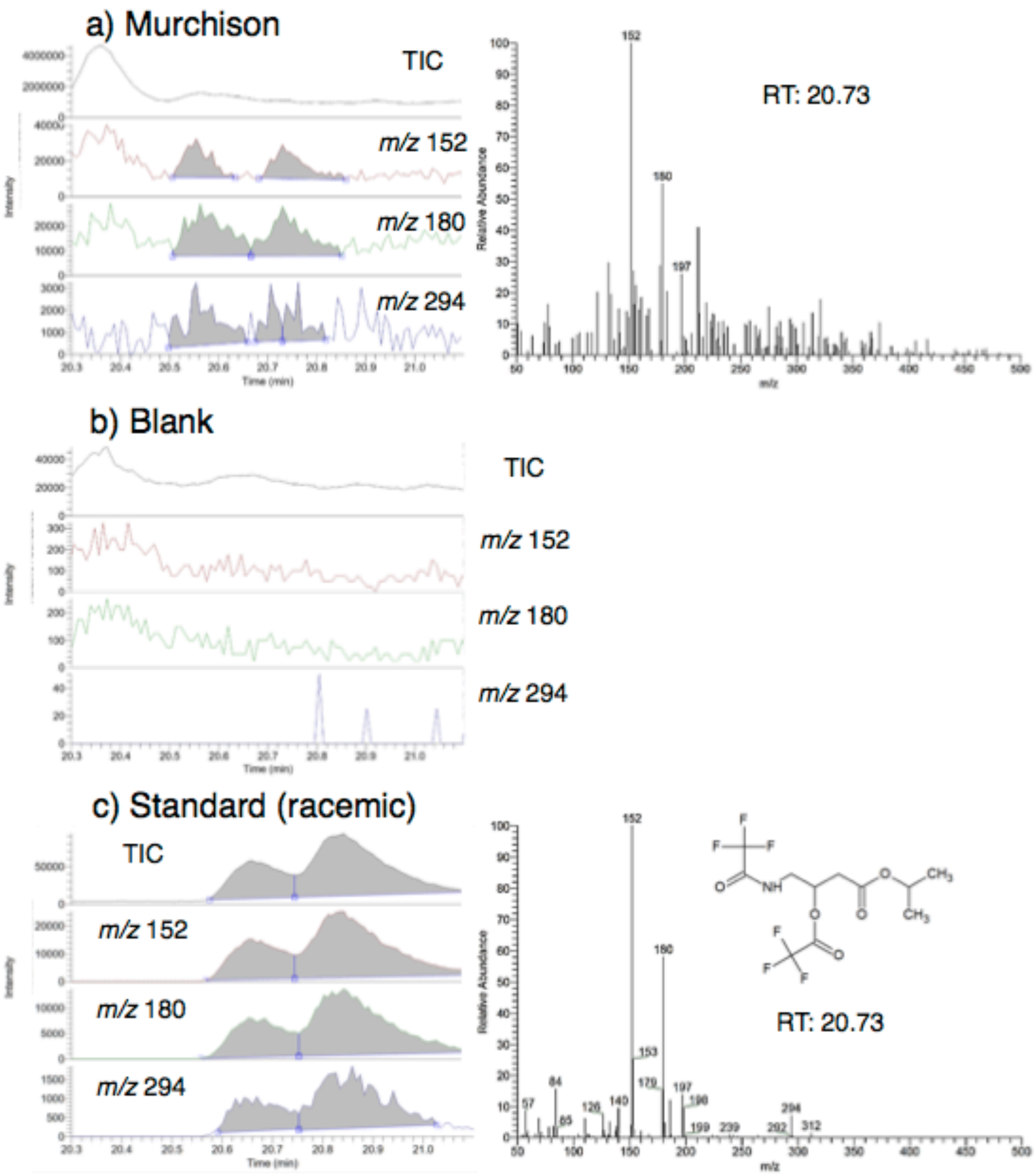

7) α-Methylserine

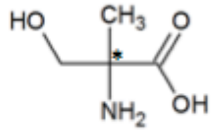

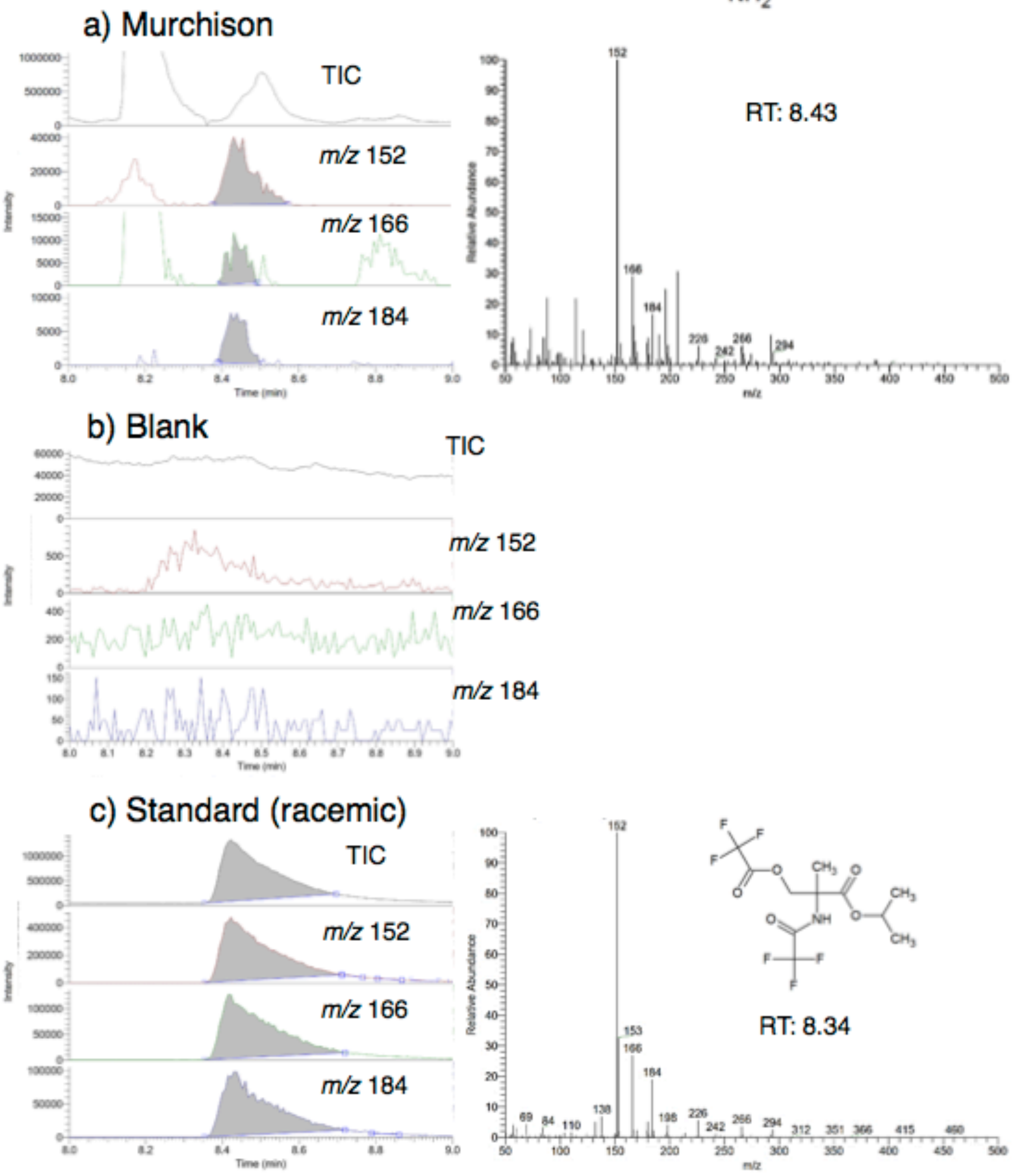

8) Isothreonine

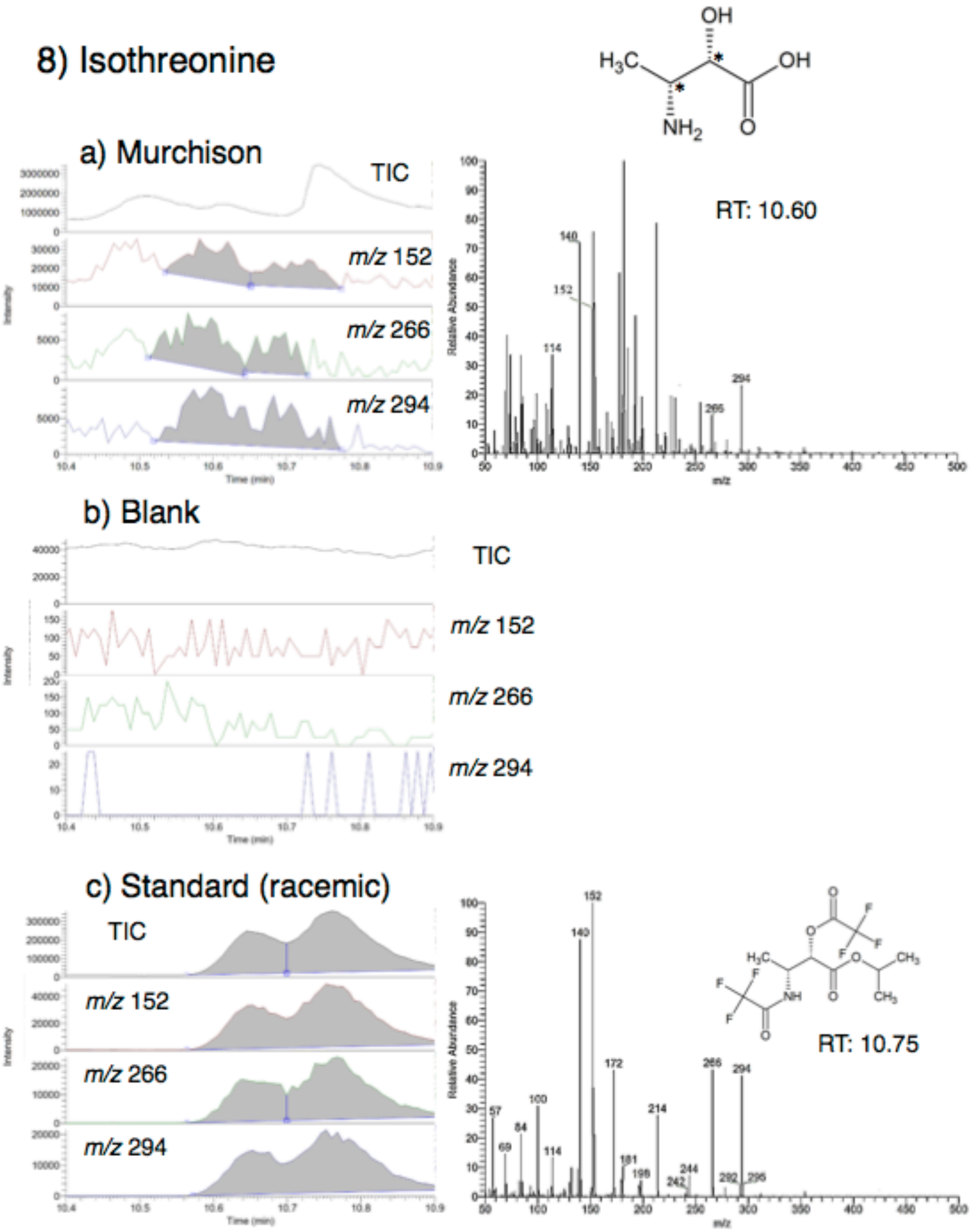

9) *allo*-Isothreonine

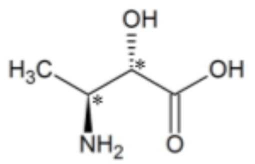

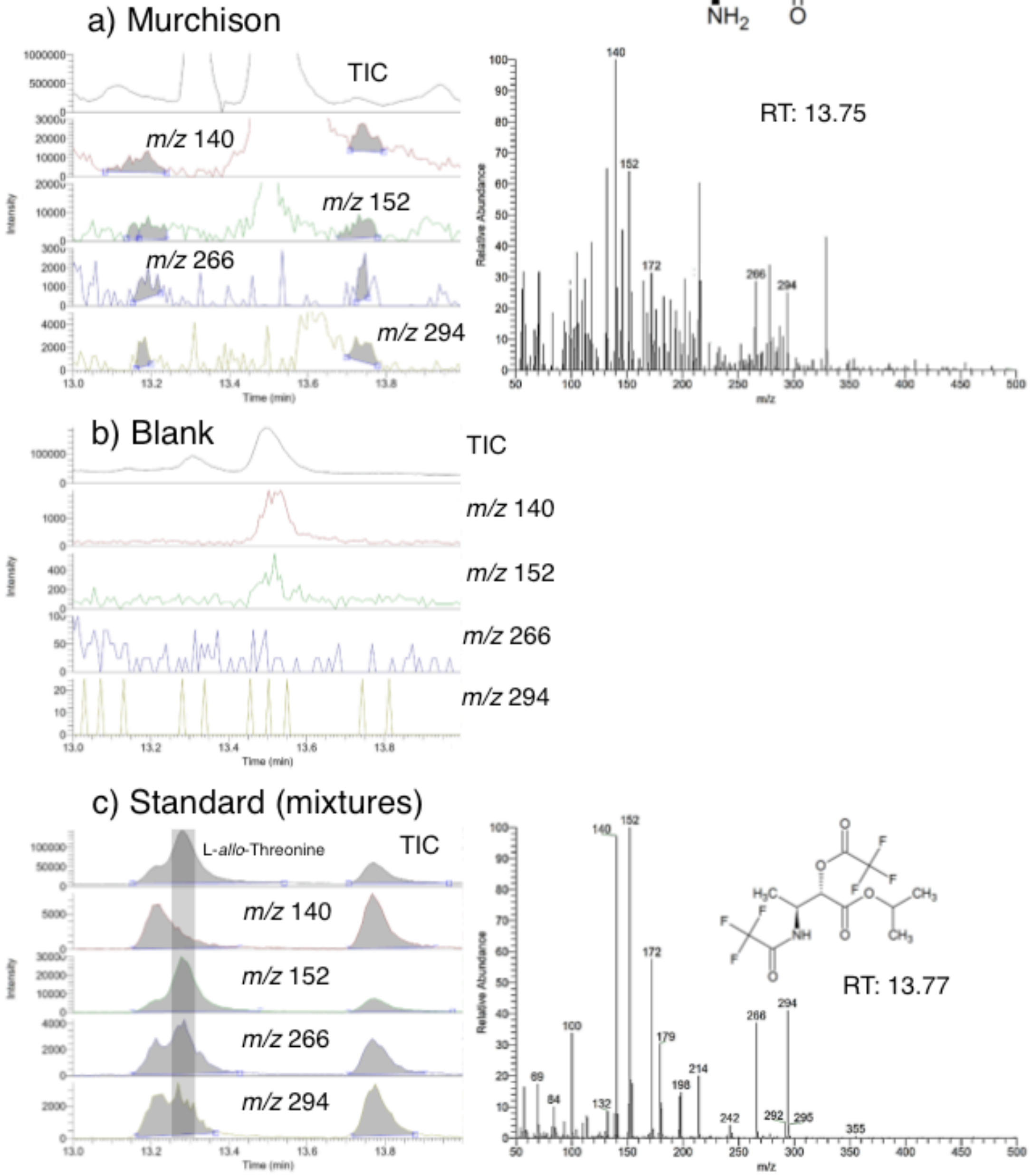

10) β-(Aminomethyl)succinic acid

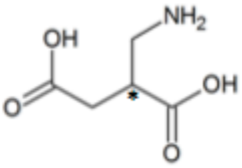

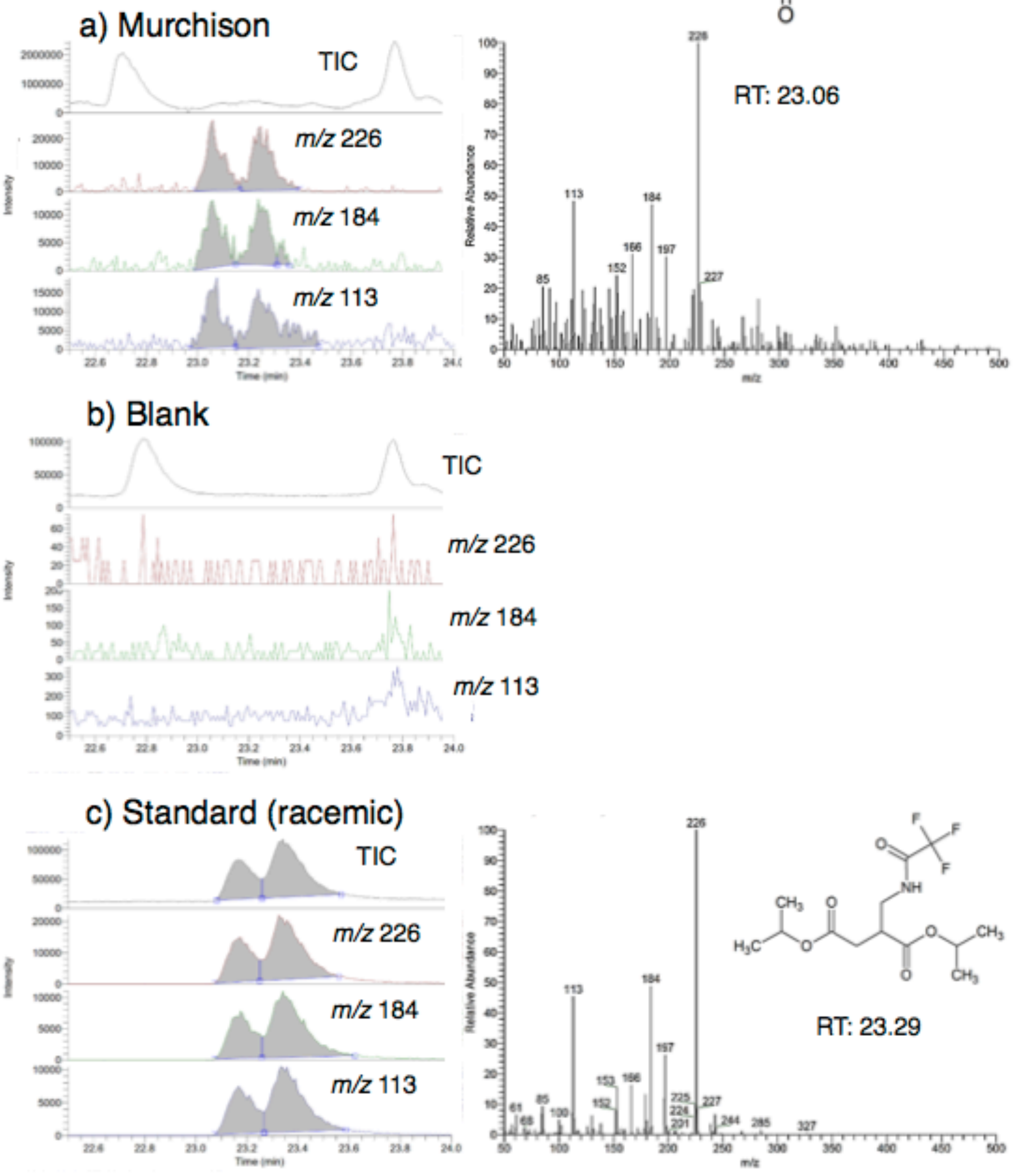

11) N-Methylserine

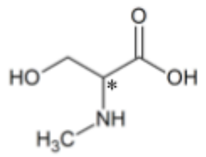

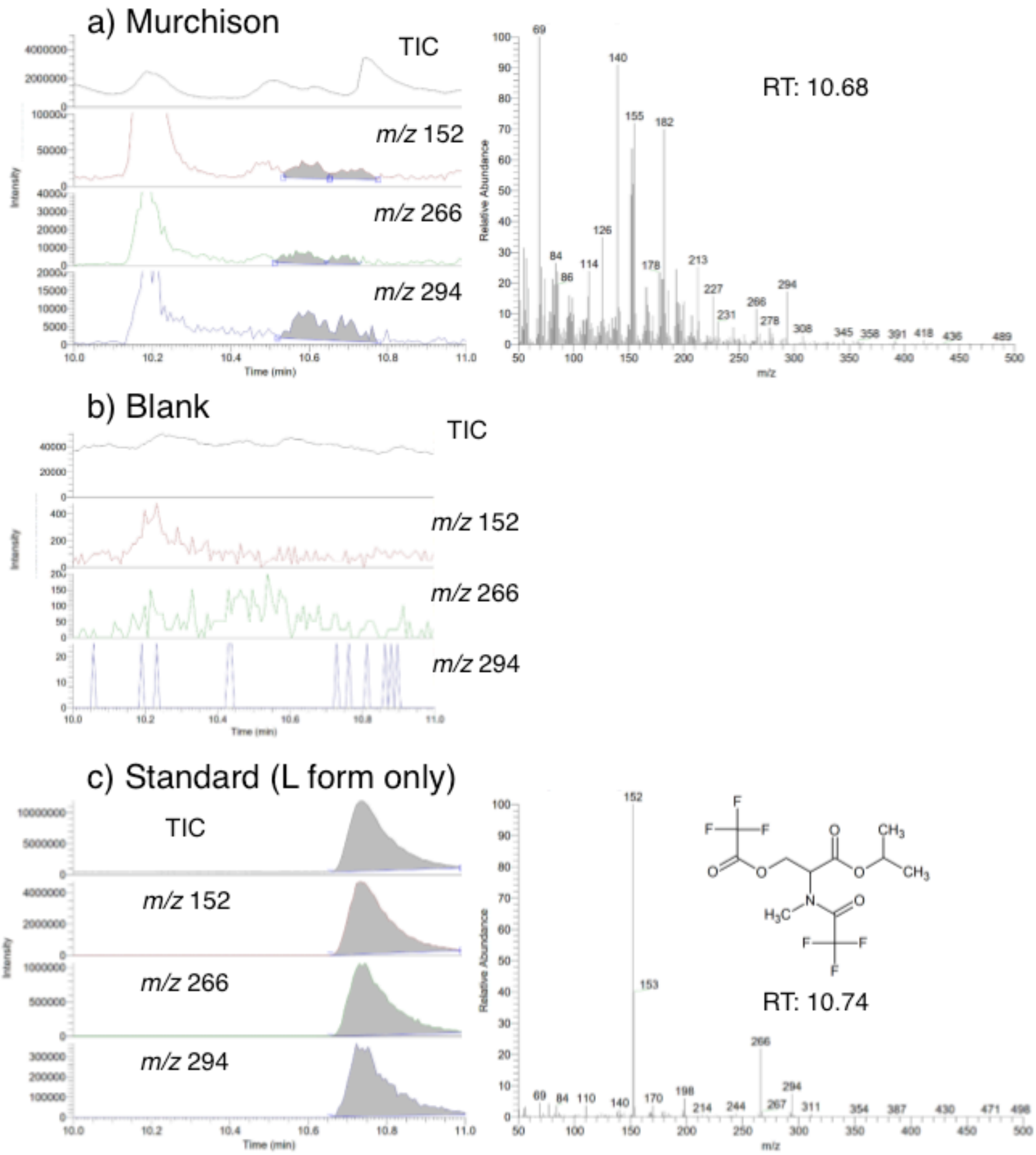

12) N-Methylisoserine

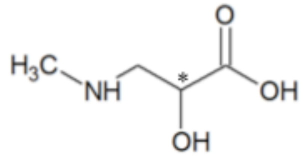

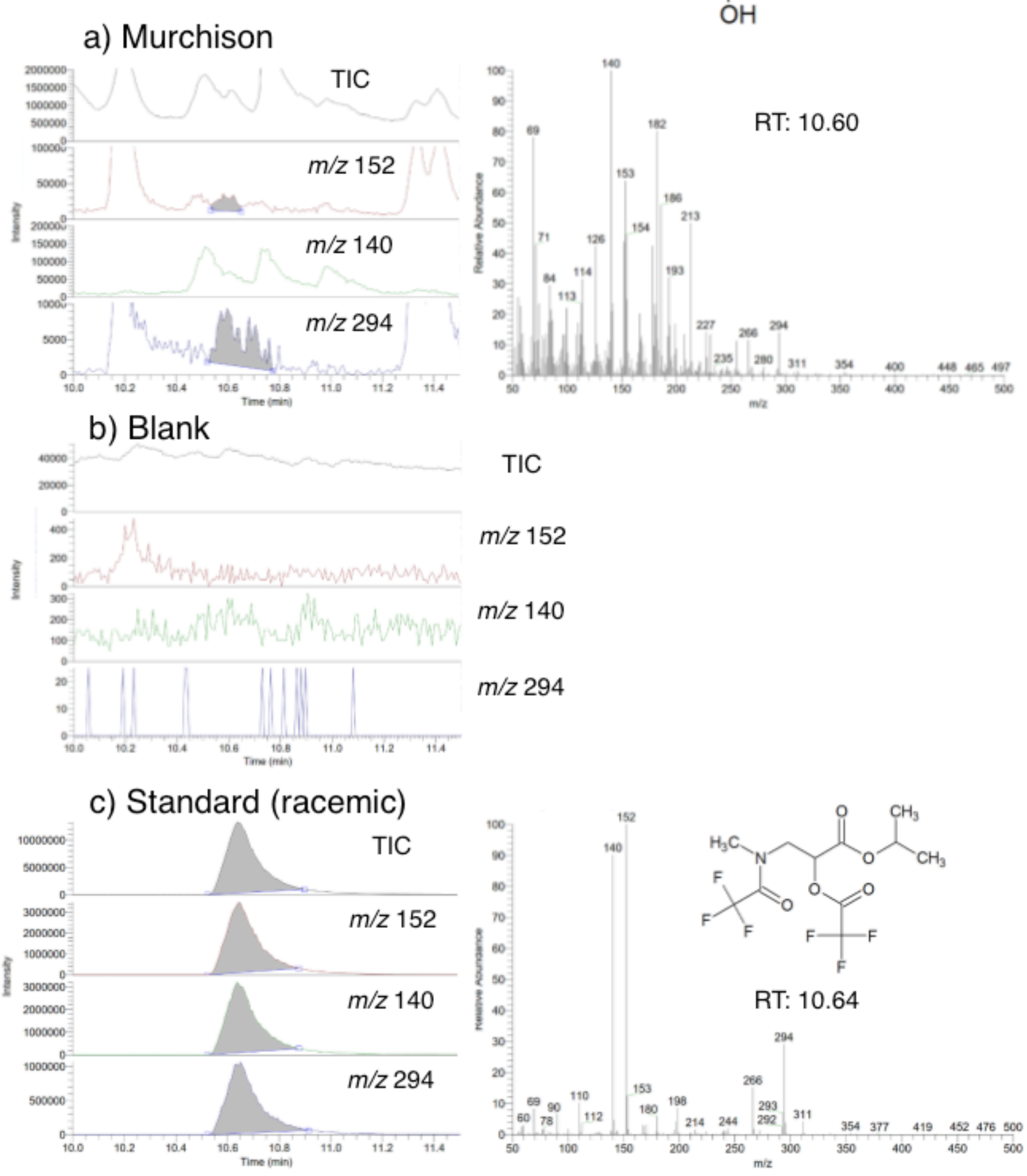

13) C_4-Hydroxy amino acid (α-Methylisoserine?)

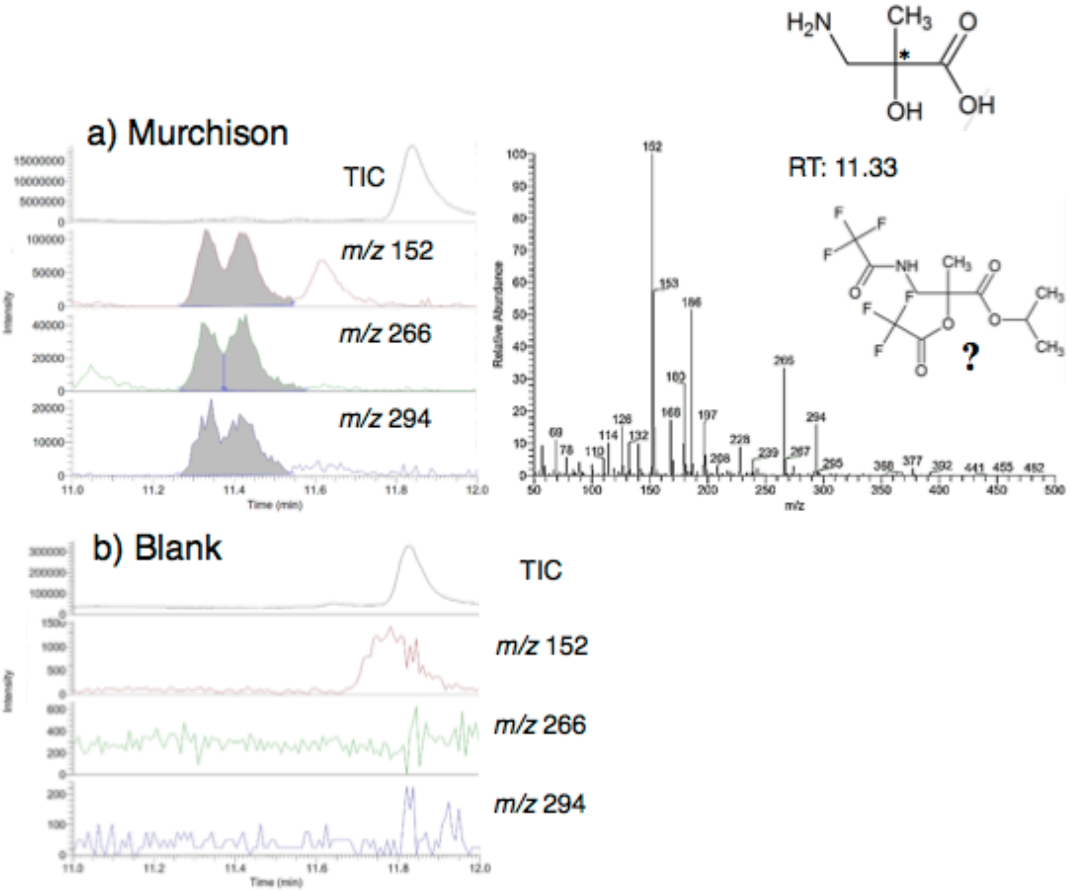

No standard available

14) C_5-Hydroxy amino acid (1)

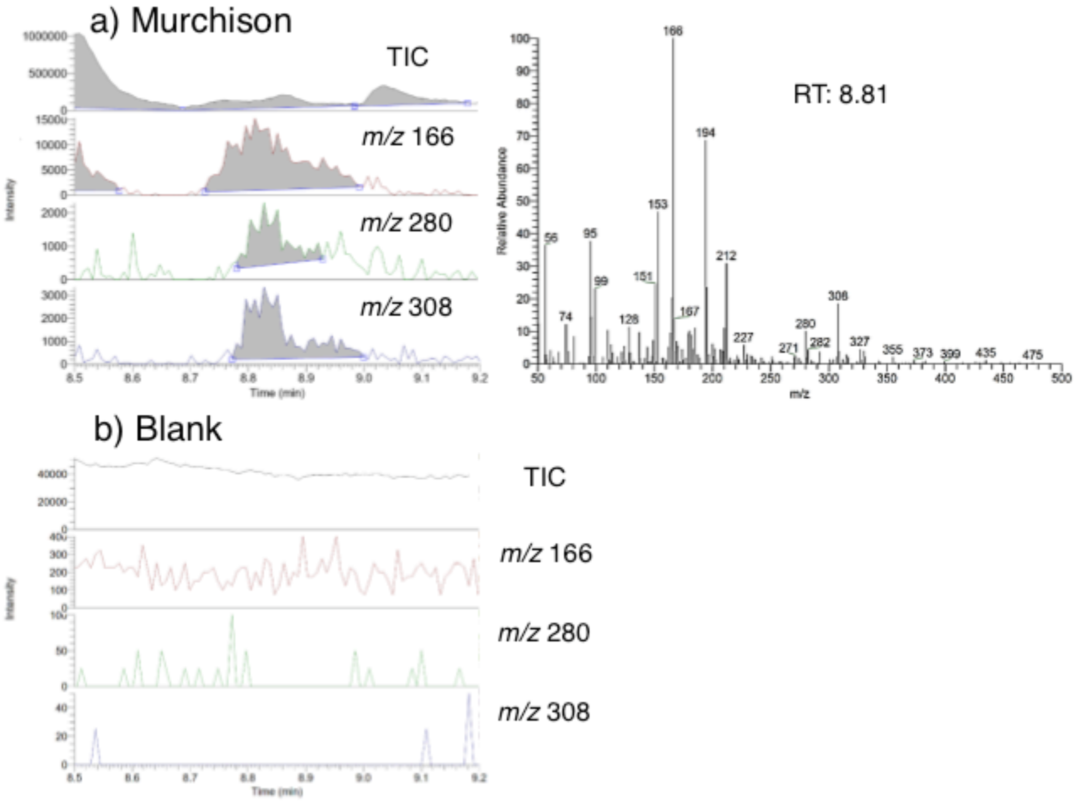

No standard available

15) C_5-Hydroxy amino acid (2)

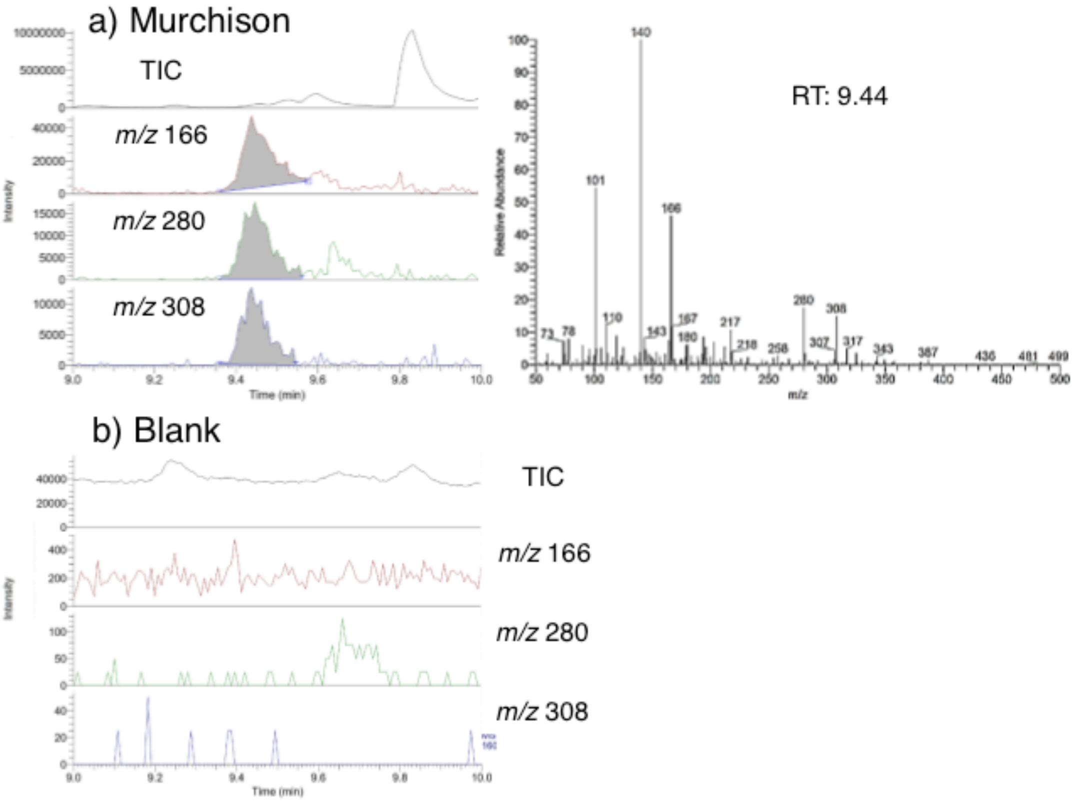

No standard available

16) C_5-Hydroxy amino acid (3)

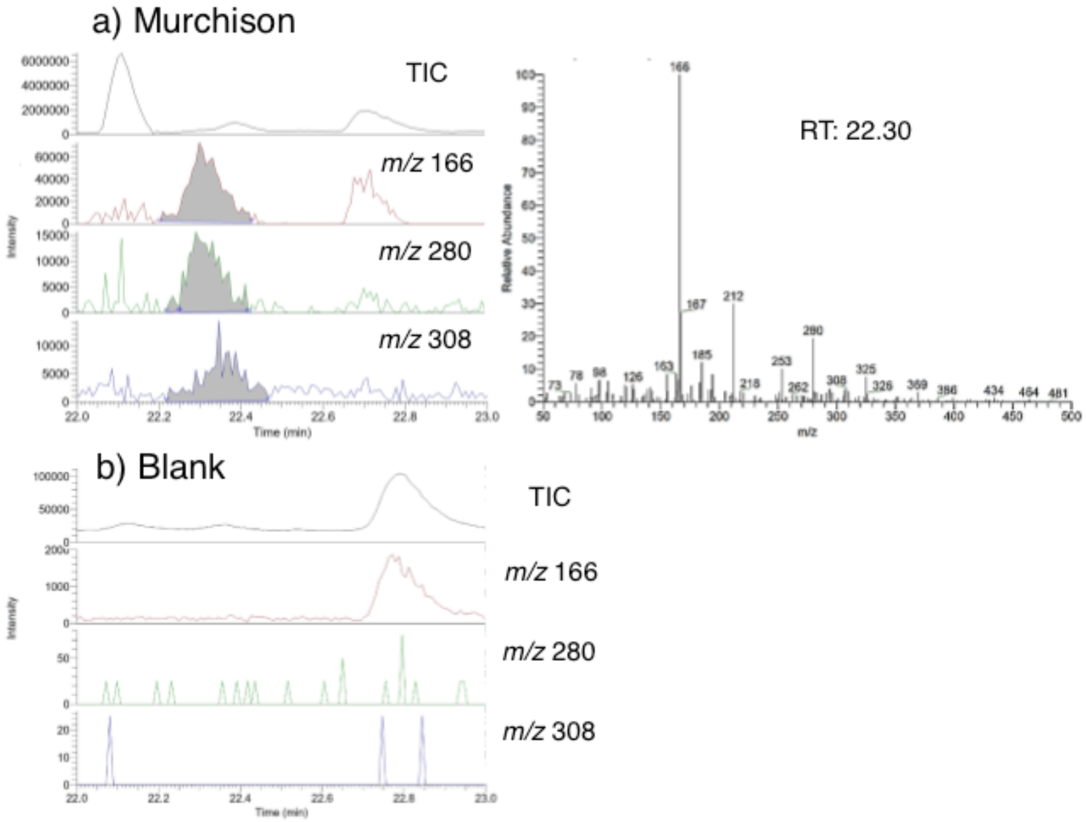

No standard available

17) Threonine

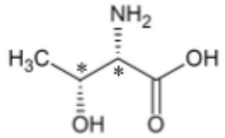

a) Murchison

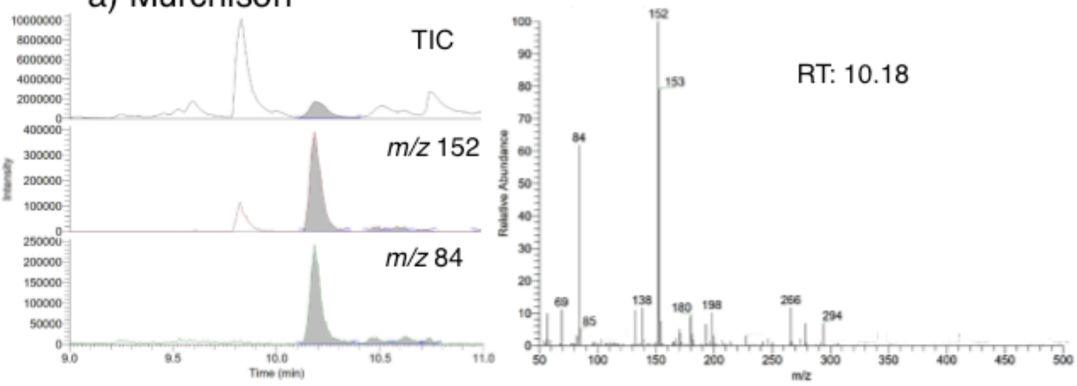

b) Blank

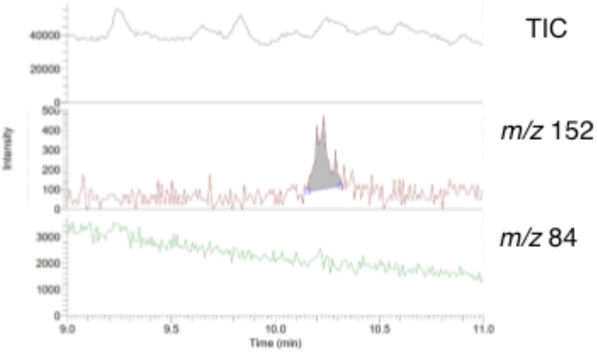

c) Standard (racemic)

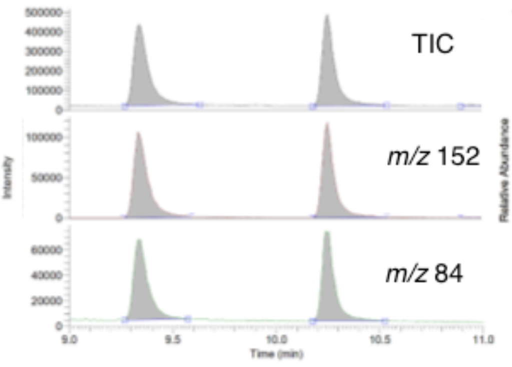

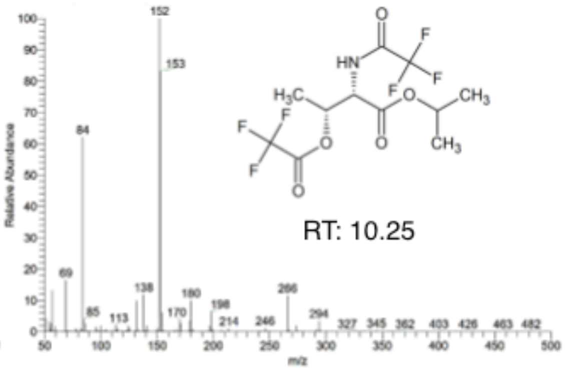

18) allo-Threonine

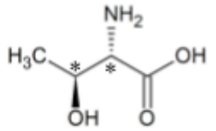

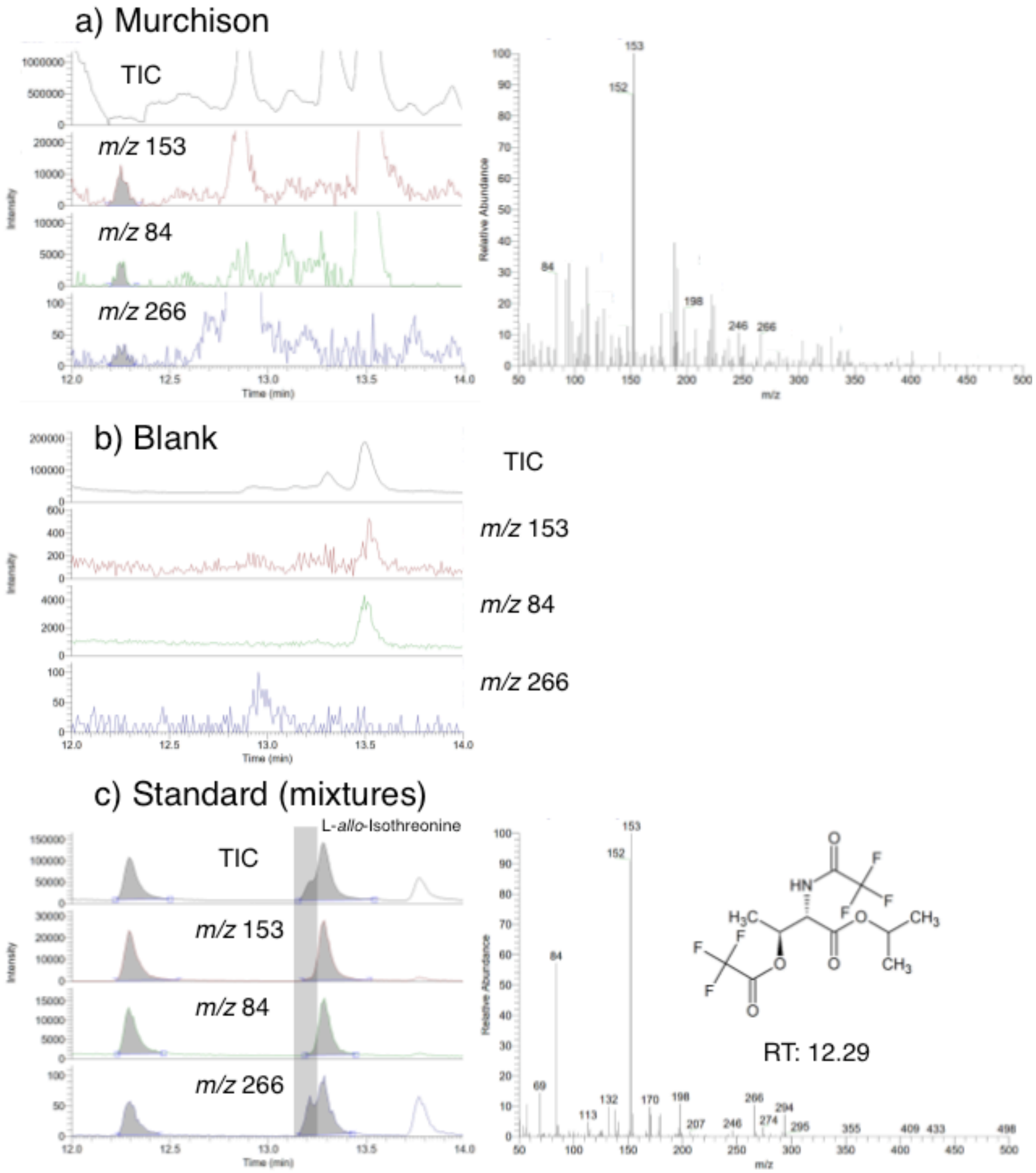

5. Mass chromatograms and mass spectra of homoserine detected in the experimental product with its standard

a) Stimulation experiment (D form, former peak)

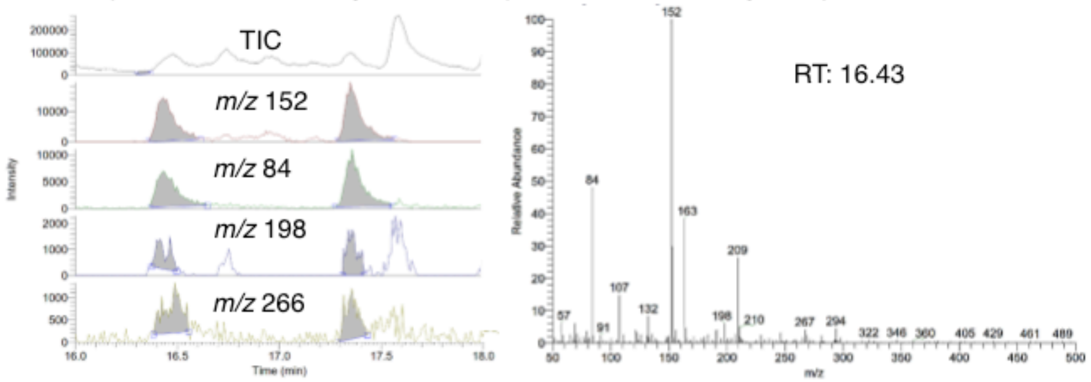

b) Stimulation experiment (L form, latter peak)

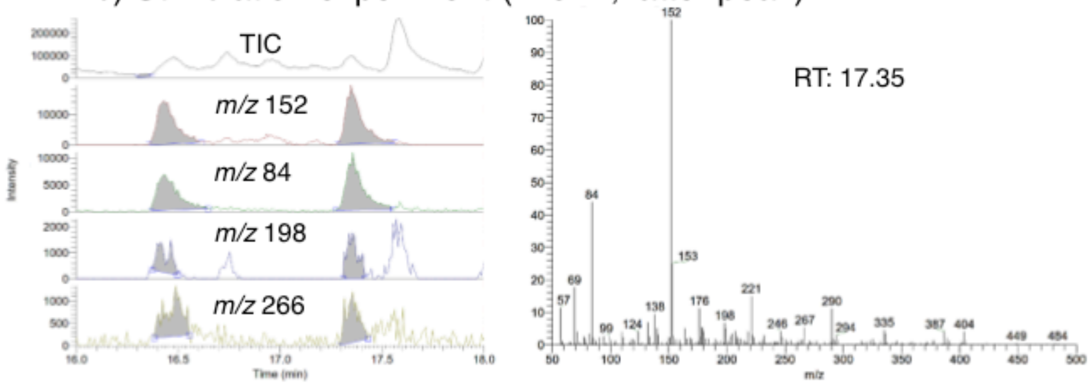

c) Standard (L form only)

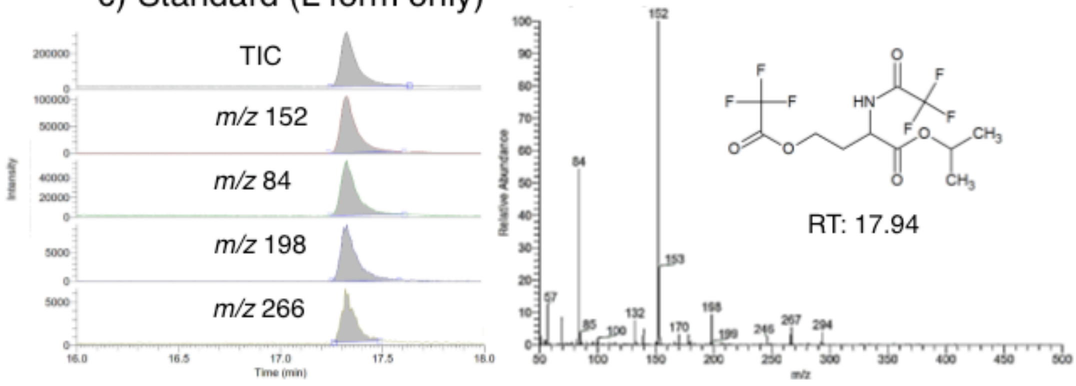

6. A possible formation pathway of 2-(aminomethyl)succinic acid

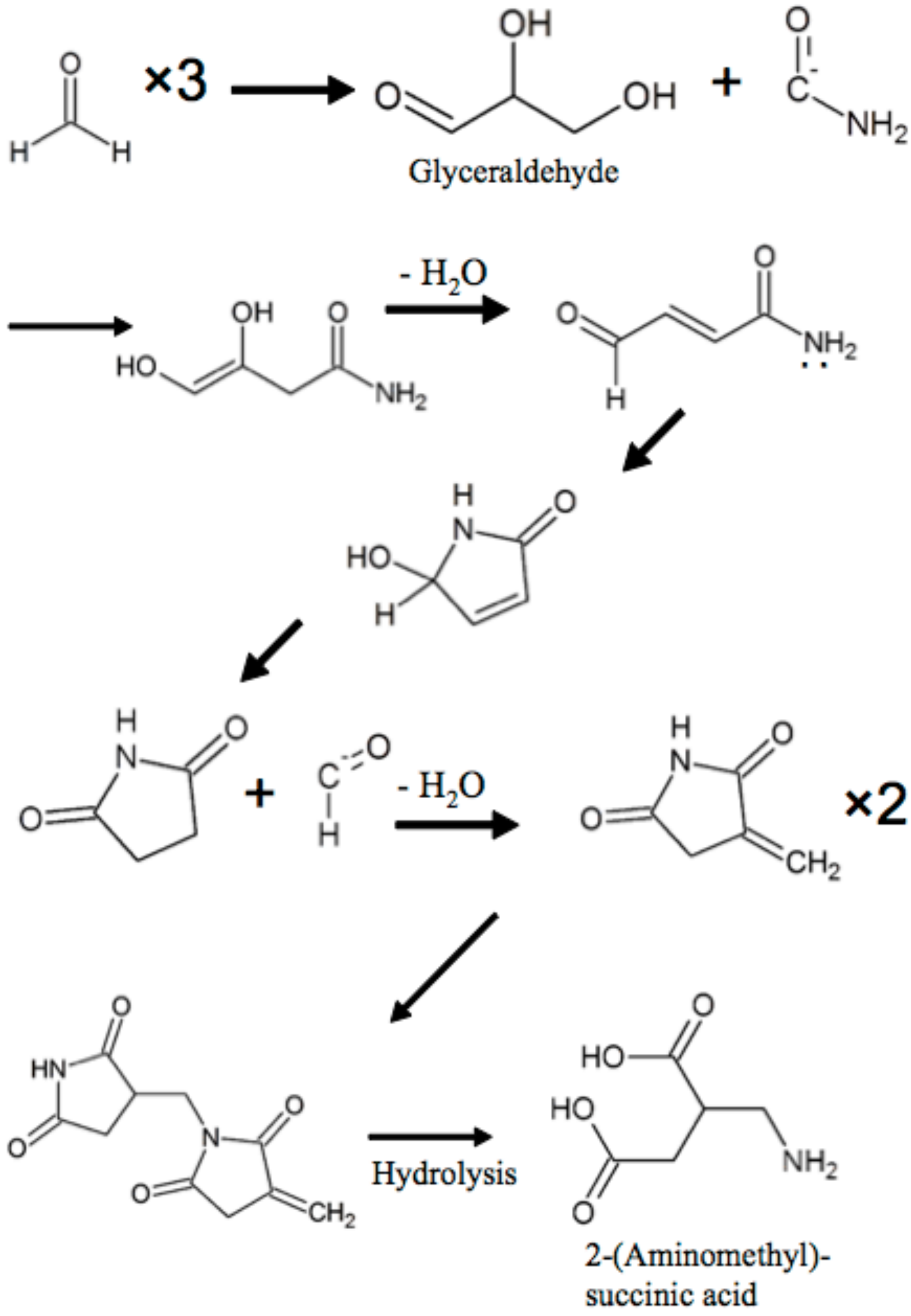

Supplement: Supplementary file 1 — Supplementary Info [file 41598_2017_693_MOESM1_ESM.pdf]
